# The cytoskeleton adaptor protein Sorbs1 controls the development of lymphatic and venous vessels in zebrafish

**DOI:** 10.1186/s12915-024-01850-z

**Published:** 2024-02-27

**Authors:** Alexandra Veloso, Anouk Bleuart, Louise Conrard, Tanguy Orban, Jonathan Bruyr, Pauline Cabochette, Raoul F. V. Germano, Giel Schevenels, Alice Bernard, Egor Zindy, Sofie Demeyer, Benoit Vanhollebeke, Franck Dequiedt, Maud Martin

**Affiliations:** 1 Interdisciplinary Cluster for Applied Genoproteomics (GIGA-R), University of Liège (ULiège), Liège, Belgium; 2 Laboratory of Gene Expression and Cancer, GIGA-Molecular Biology of Diseases, University of Liège (ULiège), Liège, Belgium; 3 Laboratory for the Molecular Biology of Leukemia, Center for Human Genetics, KU Leuven, Leuven, Belgium; 4 Center for Microscopy and Molecular Imaging, Université Libre de Bruxelles (ULB), B-6041 Gosselies, Belgium; 5 Department of Molecular Biology, Laboratory of Neurovascular Signaling, ULB Neuroscience Institute, Université Libre de Bruxelles (ULB), B-6041 Gosselies, Belgium; 6 Laboratory for Molecular Biology and Genetic Engineering, GIGA-R, University of Liège (ULiège), Liège, Belgium; 7 WEL Research Institute (WELBIO Department), Avenue Pasteur, 6, 1300 Wavre, Belgium; 8 Present Address: Laboratory of Developmental Genetics, ULB Neuroscience Institute, Université Libre de Bruxelles, B-6041 Gosselies, Belgium

**Keywords:** Sorbs1, Lymphangiogenesis, BMP signaling, Vegfc, Angiogenesis, Rho GTPases, Adhesion, Migration

## Abstract

**Background:**

Lymphangiogenesis, the formation of lymphatic vessels, is tightly linked to the development of the venous vasculature, both at the cellular and molecular levels. Here, we identify a novel role for Sorbs1, the founding member of the SoHo family of cytoskeleton adaptor proteins, in vascular and lymphatic development in the zebrafish.

**Results:**

We show that Sorbs1 is required for secondary sprouting and emergence of several vascular structures specifically derived from the axial vein. Most notably, formation of the precursor parachordal lymphatic structures is affected in *sorbs1* mutant embryos, severely impacting the establishment of the trunk lymphatic vessel network. Interestingly, we show that Sorbs1 interacts with the BMP pathway and could function outside of Vegfc signaling. Mechanistically, Sorbs1 controls FAK/Src signaling and subsequently impacts on the cytoskeleton processes regulated by Rac1 and RhoA GTPases. Inactivation of Sorbs1 altered cell-extracellular matrix (ECM) contacts rearrangement and cytoskeleton dynamics, leading to specific defects in endothelial cell migratory and adhesive properties.

**Conclusions:**

Overall, using in vitro and in vivo assays, we identify Sorbs1 as an important regulator of venous and lymphatic angiogenesis independently of the Vegfc signaling axis. These results provide a better understanding of the complexity found within context-specific vascular and lymphatic development.

**Supplementary Information:**

The online version contains supplementary material available at 10.1186/s12915-024-01850-z.

## Background

The adult circulatory system encompasses the blood and lymphatic vasculatures, which are intimately connected at the developmental, anatomical, and functional levels. In the embryo, the blood vasculature develops through a sequence of events that have been extensively characterized in the past decades [1]. Despite recent progress, our understanding of lymphangiogenesis, the formation of lymphatic vessels, lags far behind that of angiogenesis, the formation of new blood vessels. Because of its critical role in tissue homeostasis and immune surveillance, lymphangiogenesis has gained a lot of attention in recent years, leading to the identification of an increasing yet limited number of molecular drivers. Beyond these advances, recent reports also pointed to the protective and reparative therapeutic potential of enhancing lymphangiogenesis in several pathological contexts such as myocardial infarction [2–4], glioblastoma [5] and renal dysfunction [6].

As for vascular development, the zebrafish model has significantly contributed to expand our understanding of the lymphatic system formation and biology. More specifically, the stereotypical formation of the trunk lymphatic network has been extensively used to decipher the driving principles of lymphangiogenesis. Trunk lymphatic endothelial cell (LEC) precursors arise from transdifferentiation of venous ECs located within the posterior cardinal vein (PCV). Specification of LECs is triggered by the expression of the transcription factor Prox1a and is followed by their effective egression from the vein. This egression process involves asymmetric division and is under the control of cytokines and growth factors driving specific transcriptional and post-transcriptional programs [7–16]. At 32–34 h post-fertilization (hpf), dorsal sprouting of lymphatic-fated cells guided by signals originating from various cellular sources [17] contributes to the transient development of a longitudinal string of parachordal lymphangioblasts (PLs) 10 h later. At around 60 hpf, parachordal LECs start to migrate ventrally and dorsally to form the major trunk lymphatic network consisting of the thoracic duct (TD), the intersegmental lymphatic vessels (ISLVs), and the dorsal longitudinal lymphatic vessels (DLLV) [18, 19].

Illustrating the remarkable plasticity of ECs, not all the vascular sprouts emerging from the PCV and migrating dorsally alongside the artery-derived primary intersegmental vessels (aISVs) participate in building the lymphatic vessels. Approximately half of them, almost undistinguishable except for their reduced Prox1a expression, will connect and anastomose to the proximal region of aISVs to form venous ISVs (vISVs) [18, 20, 21]. Adding to the behavioral heterogeneity and specialization of the venous ECs from the PCV, ventral angiogenic sprouting also occurs from the posterior and anterior parts of the axial vein at different time points. ECs in the caudal region sprout from the floor of the caudal vein (CV) at around 27 hpf and migrate towards the ventral side of the embryo to form the caudal vascular plexus (CVP), a distinctive fenestrated network of vessels [22]. More rostrally, formation of the subintestinal venous plexus (SIVP), which will eventually provide blood supply to the digestive tract, starts at around 30 hpf. This ventral migration process leads to the formation at 3 days post-fertilization (dpf) of a basket-like plexus composed of vertical interconnecting vessels (ICVs) that drain into a transversal subintestinal vein (SIV) [23–25].

Studies of mutants isolated from forward genetic screens or associated with human diseases led to the establishment of the Vegfc/Flt4 axis as the central pathway for lymphangiogenesis [26–29]. Accordingly, the currently growing list of trunk lymphangiogenesis regulators almost exclusively relates to molecules involved in Vegfc/Flt4 signaling, some of them acting directly upstream such as CCBE1 [30–33] or the transcription factor HHEX [9] or downstream like the transcription factors Mafba [34] and Yap1 [35]. Acting through multiple intracellular events, including the activation of the common effector of Vegf receptors Erk [7], Vegfc signaling controls several aspects of lymphangiogenesis including LEC differentiation through Prox1 expression, proliferation, and migration after cell cycle arrest [36]. Whereas these signaling cues seem to act indistinctly on dorsal lymphatic and venous sprouting that occur concomitantly, with the majority of effectors impacting on both lymphatic vessel and vISV formation [26, 31, 37, 38], ventral angiogenesis of the caudal vascular and subintestinal plexus relies on BMP signaling [22, 24].

Our understanding of angiogenic cues that drive formation of specific vascular beds is only emerging. Even less is known about the intracellular components that define specific endothelial cell behavior during establishment of highly conserved organ-specific vascular patterns. Sorbs1 (Cbl-associated protein CAP/ponsin) belongs to the SoHo family of adaptor proteins that includes two other members, Sorbs2 (Arg-binding protein 2, ArgBP2) and Sorbs3 (Vinexin). Early following their discovery, SoHo proteins were shown to localize to various actin-based structures, including z-discs, stress fibers, cell-ECM and cell–cell adhesions [39–45]. Sorbs1 interactions with several structural and signaling cytoskeletal components, such as vinculin and paxillin, strengthened the idea that it might function as an adaptor protein coordinating multiple signaling complexes regulating the actin cytoskeleton [46, 47]. In agreement with these observations, in vitro studies showed that Sorbs1 and the other family members are important regulators of actin-dependent processes, such as migration, adhesion, and mechano-transduction [45, 48, 49]. These cytoskeleton-based processes are essential to support and control the morphogenic events that endothelial cells have to go through during blood and lymphatic vessel formation [50].

Here, we report that Sorbs1 is important for angiogenesis and lymphangiogenesis. Using a combination of in vivo and in vitro approaches, we demonstrate that Sorbs1 controls EC adhesion signaling through modulation of specific RhoGTPases activities and consequently participates in the formation of specific venous and lymphatic structures originating from the main axial vein, both dorsally and ventrally. The spatiotemporal pattern of Sorbs1 expression is consistent with these functions. Surprisingly, despite its major impact on trunk lymphatic structures, Sorbs1 is not involved in Vegfc pathway but appears to participate in BMP signaling.

## Results

### Sorbs1 genetic depletion is associated with pericardial edema formation

**Fig. 1:**
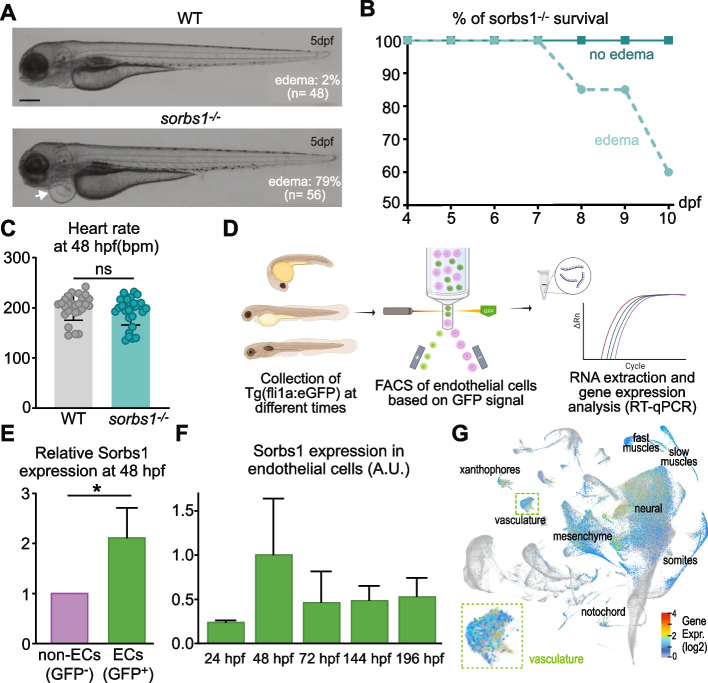
*Sorbs1* expression is enriched in the endothelium and its deletion in zebrafish results in cardiac edemas. **A** Transmitted light images of live wild-type (WT) and sorbs1 mutant (*sorbs1*^*−/−*^) zebrafish embryos at 5 days post-fertilization (dpf). The white arrow indicates an example of edema observed in *sorbs1*^*−/−*^ embryo. Proportions of embryos with edemas in each genotype are indicated. Scale bar represents 250 μm. **B** Quantification of the percentage of survival for *sorbs1*^*−/−*^ embryos presenting or not edemas from 4 to 10 dpf (*n* = 23 and *n* = 47, respectively). **C** Quantification of the heart rate in WT and *sorbs1*^*−/−*^ embryos at 48 hpf (*n* = 45 and *n* = 49, respectively). **D** Illustration of the procedure used to sort endothelial cells by FACS (fluorescence-activated cell sorting) from *Tg(fli1a:eGFP)y1* zebrafish embryos, at different stages of development, followed by RNA extraction and reverse transcription-qPCR (RT-qPCR). **E**, **F** RT-qPCR analysis of *sorbs1* expression relative to Elfa after FACS sorting as described in **D**, in endothelial cells (ECs, GFP + , green bars) vs non-endothelial cells (non-ECs, GFP-, purple bar) (**E**) or in endothelial cells at different time points of embryonic development (**F**) (*P < 0.05, unpaired *t*-test). **G** UMAP of *sorbs1* expression at single-cell level obtained from the Daniocell database consisting of whole-animal wild-type zebrafish across multiple stages of development (14–120 hpf). Cluster annotation was taken from the Daniocell resource

To investigate the function of Sorbs1 in vivo, we took advantage of the zebrafish model. We performed phylogenetic analysis using results from BLAST homology searches against NCBI and Ensembl databases and identified SoHo family orthologs in zebrafish. A single *sorbs1* ortholog (ENSDARG00000103435), two *sorbs2* orthologs, *sorbs2a* (ENSDARG00000003046) and *sorbs2b* (ENSDARG00000061603), and a single *sorbs3* ortholog (ENSDARG00000037476) were identified (Additional file 1: Figure S1A). To assess the role of Sorbs1 in zebrafish development, we used the CRISPR/Cas9 system to generate a *sorbs1* mutant allele. We selected F1 heterozygous carriers with a 14 base pair (bp) deletion at codon 178, within the SoHo domain. This mutation generates a premature stop codon at codon 182 (Additional file 1: Figure S1B). Homozygous mutants (referred to as *sorbs1*^*−/−*^) from heterozygous in-crosses did not express any detectable full-length sorbs1 protein (Additional file 1: Figure S1C). A large proportion of the *sorbs1*^*−/−*^ larvae exhibited large edemas around the heart and the intestinal tract, clearly visible at 5 dpf (Fig. 1A) and strongly impacting on the viability of the embryos with about 40% of edema-developing embryos dying within 10 dpf (Fig. 1B). As *sorbs1* mutants displayed overall normal morphogenesis (Additional file 1: Figure S1D) and cardiac function (Fig. 1C), we suspected that these edemas could be indicative of vascular and/or lymphatic defects. In agreement with a potential vascular function, Sorbs1 protein expression was detected in blood vessel endothelium by immunohistochemical analysis of various adult quiescent human tissues (Additional file 1: Fig. S1E, black arrows). In addition, Sorbs1 protein was detected in a series of cultured human ECs, with the highest levels being observed in venous and lymphatic ECs (Additional file 1: Fig. S1F). In zebrafish, whole mount in situ hybridization revealed a ubiquitous expression of *sorbs1* throughout development (Additional file 1: Fig. S1G). To validate *sorbs1* expression in the zebrafish vascular endothelium, we used the *Tg(fli1a:eGFP)y1* transgenic line, in which lymphatic, arterial, and venous ECs express green fluorescent protein (GFP), and sorted GFP-positive cells and GFP-negative cells by flow cytometry (Fig. 1D). Quantitative PCR analysis revealed that expression of *sorbs1* was detectable and significantly higher in GFP-positive cells, as compared to GFP-negative cells (Fig. 1E). Although expression of Fli1a is not entirely restricted to ECs, these results indicate that *sorbs1* might be more expressed in EC than in non-ECs. The expression of *sorbs1* in ECs was maximal at around 48 hpf, when active lympho-venous sprouting is occurring (Fig. 1F). To confirm the expression of *sorbs1* in the endothelial compartment, we analyzed publicly available single-cell RNA sequencing data of 3.3 to 120 hpf zebrafish from the Daniocell resource [51]. These data revealed a non-exclusive but enriched expression of *sorbs1* in the vasculature (Fig. 1G).

### Sorbs1 is important for lymphangiogenesis in zebrafish

**Fig. 2:**
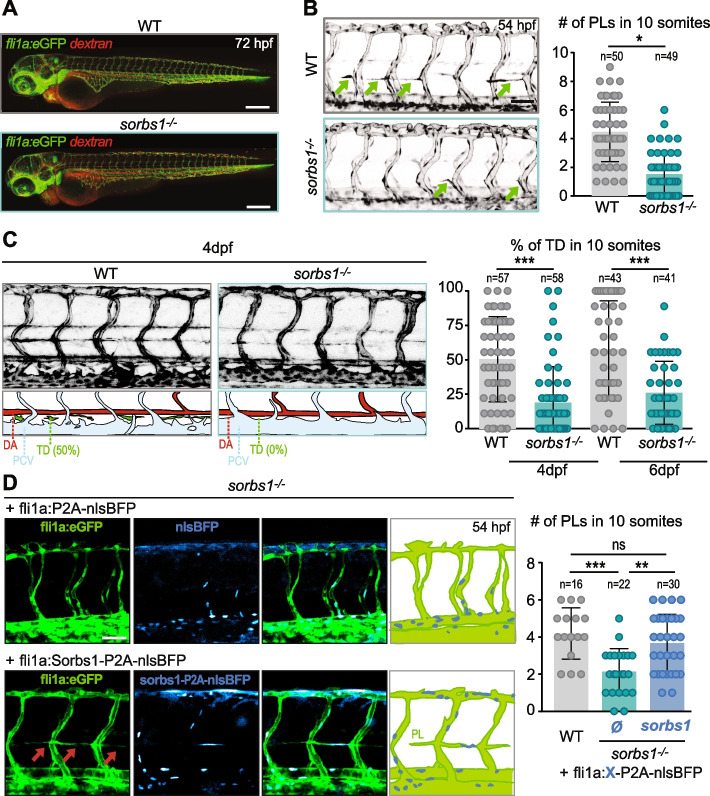
Sorbs1 is necessary for trunk lymphangiogenesis in vivo. **A** Confocal microscopy (Z-maximum intensity projections) of 72 hpf WT and *sorbs1*^*−/−*^* Tg(fli1a:eGFP)y1* embryos vasculature (green) after injection of tetramethylrhodamine dextran (2,000,000 kDa, 25 μg/μl) in the circulation to assess vessel perfusion (red). Scale bar represents 250 μm. **B** Confocal microscopy (Z-maximum intensity projections) of the trunk vasculature of WT and *sorbs1*^*−/−*^* Tg(fli1a:eGFP)y1* embryos at 54 hpf used to quantify the number of parachordal lymphangioblasts (PLs) (green arrows) over 10 somite segments. Scale bars represent 50 μm (*n* = number of embryos, respectively; *P < 0.05, Mann–Whitney *U*-test). **C** Z-maximum projections of confocal images of the trunk vasculature from 4 dpf *Tg(fli1a:eGFP)y1* WT or *sorbs1* knock-out embryos. Schematic representations of arterial (red), venous (light blue), and lymphatic (green) vessels are shown below. Dorsal aorta (DA), posterior cardinal vein (PCV), thoracic duct (TD). Scale bars represent 50 μm. Graph shows the quantification of the thoracic duct (TD) extent over 10 segments at 4 and 6 dpf in WT and *sorbs1*^*−/*^.^*−*^ embryos (*n* = number of embryos; ***P < 0.001; Mann–Whitney *U*-test). **D** Z-maximum projections of confocal images of the trunk vasculature of 54 hpf *Tg(fli1a:eGFP)y1 sorbs1* knock-out embryos expressing transgenic endothelial constructs coding for human Sorbs1 or not were used to quantify the number of PLs in 10 somites. BFP is used as transgenesis marker. Scale bar represents 50 μm (*n* = number of embryos, ns = non-significant; ***P < 0.001; **P < *0.01*; Mann–Whitney *U*-test)

To explore if the presence of edemas in *sorbs1*^*−/−*^ larvae could relate to lymphatic vascular defects, we performed microscopic observation of the vasculature of *sorbs1* mutants in the *Tg(fli1a:eGFP)y1* transgenic background. Whereas mutants showed normal morphogenesis, patterning, and lumenization of the cranial and trunk primary vasculature (Fig. 2A, Additional file 1: Figure S2A), development of lymphangiogenic structures was severely affected (Fig. 2B, C). The formation of the PLs at the horizontal trunk septum was strongly impaired: quantification analysis at 54 hpf confirmed that the proportion of somite segments with detectable PLs was significantly reduced in *sorbs1*^*−/−*^ embryos, with PLs being totally absent in approximately one third of the embryos (Fig. 2B). At approximately 60 hpf, PLs migrate ventrally from the horizontal myoseptum to form the TD (3–6 dpf), the major lymphatic trunk vessel situated between the DA and PCV. Because *sorbs1* mutants had a lower number of PLs, we reasoned that they might also exhibit defects in TD formation. To test this, we measured the length of visible TD portions in 10 somites at 4 and 6 dpf and expressed it as a percentage of the total length of the corresponding trunk segment (Fig. 2C) [52]. In control larvae, the observed length of the TD at 4 dpf represented approximately 49% of the trunk total length, a proportion that increased up to 60% at 6 dpf. Formation of the TD was greatly impaired in *sorbs1*^*−/−*^ larvae, as TD length corresponded to only 19% and 26% of the 10-somite length at 4 and 6 dpf, respectively. In a large proportion of *sorbs1*^*−/−*^ larvae (41%, 24/58), the TD was totally absent at 4 dpf, while only 14% (8/57) of control embryos had no detectable TD. As expected, the most affected *sorbs1*^*−/−*^ embryos (i.e., embryos with less than 20% of visible TD) had reduced life span (Additional file 1: Figure S2B).

As a complementary approach, we used a splice-blocking antisense morpholino (*sorbs1* Mo) targeting the exon 3/intron 3 boundary of *sorbs1*. This morpholino efficiently altered splicing with skipping of exon 3 and reduced Sorbs1 protein levels when injected at 5 ng/embryo (Additional file 1: Figure S2C, D). Similarly to *sorbs1* mutants, the vast majority of morphant embryos exhibited PL and TD defects (Additional file 1: Figure S2E, F). The specificity of our approach was confirmed by a dose dependency of the effects of the morpholino as well as when using an additional ATG blocking morpholino (Additional file 1: Figure S2D-F). The observed defects in PL and TD formation strongly suggest that Sorbs1 is important for early lymphatic development. In agreement with the idea of an endothelial function for Sorbs1, PL formation defects in *sorbs1*^*−/−*^ mutants and morphants were rescued by ectopic expression of human Sorbs1 (hSorbs1) using the pan-endothelial *fli1a* promoter or the more endothelial restricted *kdrl* promoter (Fig. 2D, Additional file 1: Figure S2G).

### Lack of Sorbs1 impairs secondary sprouting from the PCV

**Fig. 3:**
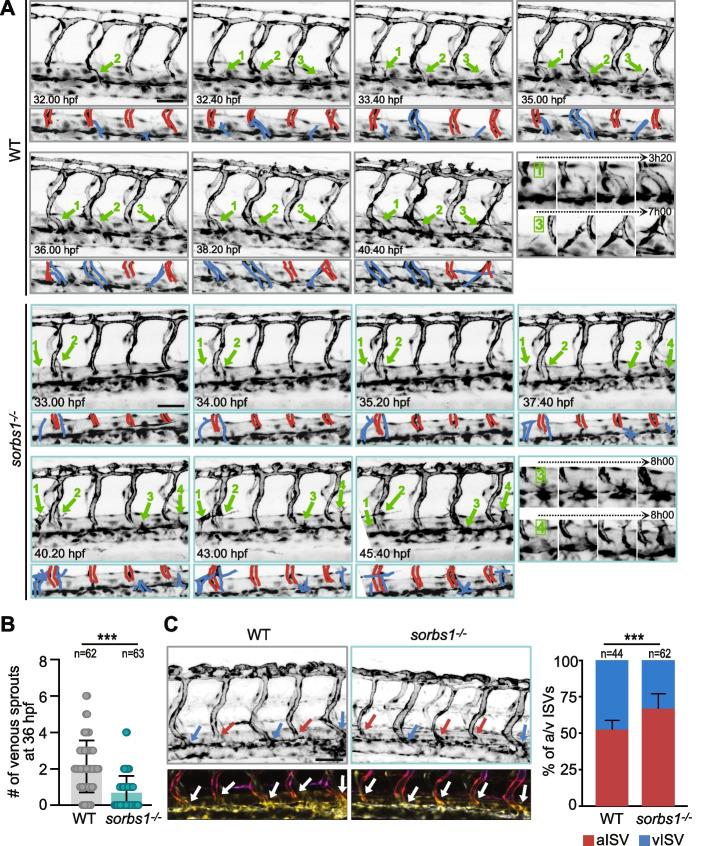
Secondary sprouting is impaired in the absence of *sorbs1*. **A** Frames (Z-maximum projections) from time-lapse confocal imaging of WT (gray boxes) and *sorbs1*^*−/−*^ (light blue boxes) *Tg(fli1a:eGFP)y1* embryos during venous secondary sprouting. Scale bar represents 50 μm. Numbered green arrows indicate sprouting events from the PCV, some of them being illustrated with zooms spanning the time indicated with the dashed arrows in the last boxes. **B** Quantification of secondary sprouts visible at 36 hpf in WT and *sorbs1* mutants (*sorbs1*^*−/−*^) (*n* = number of embryos, ***P < 0.001; Mann–Whitney *U*-test). **C** Z-maximum projections of confocal images of the trunk vasculature of 48 hpf *Tg(fli1a:eGFP)y1* WT and *sorbs1* knock-out embryos. Blue and red arrows point to venous and arterial ISVs respectively and 3D color-coded stacks are shown at the bottom. Scale bar represents 50 μm. Graph shows the quantification of percentage of aISVs and vISVs at 48 hpf (*n* = number of embryos, *** *P* < *0.001*; *χ*.^2^ with Yates correction)

PL formation starts with the dorsal migration of venous ECs from the PCV at 30–33 hpf. These venous sprouts will either form lymphatic PLs or connect to an aISV switching it to a vISV. To evaluate the role of Sorbs1 in this process, we followed the nascent secondary sprouts emerging from the PCV, i.e., sprouts not yet fused to a primary ISV or stabilized to form PL (arrows in Fig. 3A). Whereas control venous sprouts quickly interact with neighboring aISVs before either stabilizing their connection (arrows 1 and 2 in Fig. 3A, WT, Additional file 2: Movie 1) or starting to form PL [21] (arrow 3 in Fig. 3A, WT, Additional file 2: Movie 1), mutant sprouts slowly and rarely connect to aISVs. They are sometimes destabilized (arrows 1 and 3 in Fig. 3A, *sorbs1*^*−/−*^, Additional file 3: Movie 2) or hardly form at all (Additional file 1: Figure S3A, Additional file 4: Movie 3). A snapshot analysis at 36 hpf revealed that the number of sprouts was significantly reduced in *sorbs1*^*−/−*^ Tg*(fli1a:eGFP)y1* embryos compared to controls (Fig. 3B) and that the venous sprouts that eventually connect with the neighboring aISV in *sorbs1*^*−/−*^ embryos take more time to do so than in WT embryos (Additional file 1: Figure S3B). These defects in secondary sprouting were confirmed by looking at *sorbs1* morphants (Additional file 1: Figure S3C). Because approximately half of the secondary sprouts gives rise to PLs, the defective PCV secondary sprouting in the absence of Sorbs1 could explain the reduced number of PLs. To assess if it also affected the migration of the alternatively fated venous sprouts (i.e., the sprouts that will remodel the primary aISVs into vISVs), we counted the number of vISVs, i.e., ISVs connected to the PCV over a 10-somite region at 48 hpf. In wild-type embryos, approximately half of the ISVs were connected to the PCV and thus scored as of venous identity. By contrast, the proportion of vISVs was significantly lower (35.5%) in *sorbs1* mutant embryos (Fig. 3C). A similar reduction in vISVs was also observed in *sorbs1* morphants (Additional file 1: Figure S3D). These observations suggest that Sorbs1 knockout/knockdown affects the secondary wave of migrating ECs from the PCV, which is associated with both lymphatic and vISV network formation.

### Sorbs1 expression is enriched in venous tissues

**Fig. 4:**
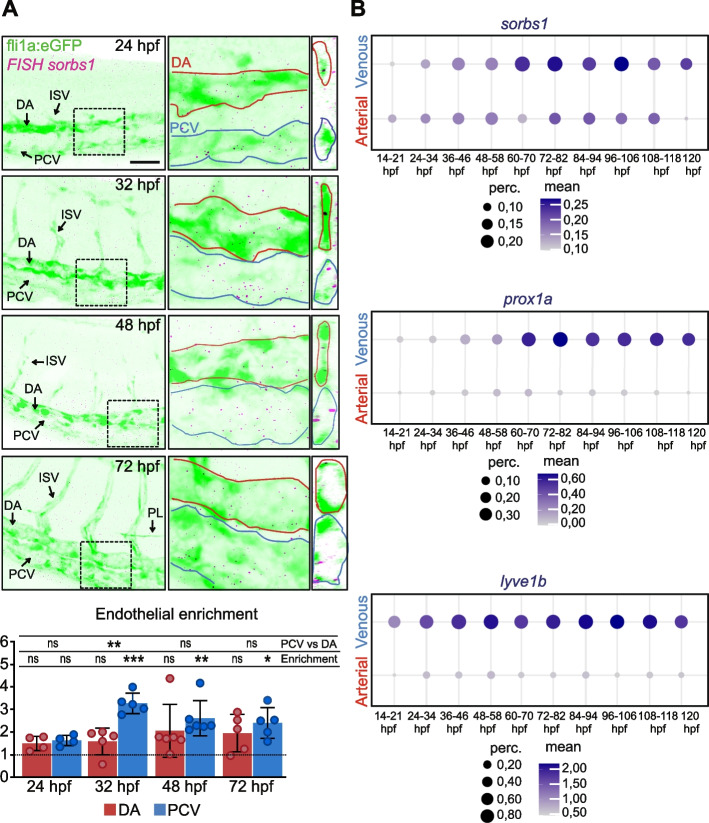
*Sorbs1* is expressed in the vasculature throughout development. **A** Z-maximum projections of confocal images of the trunk vasculature of WT embryos at the indicated time of development and stained for *sorbs1* transcripts (magenta dots) and for vessels (gfp immunostaining, green) using FISH. The DA and PCV are delineated in red and blue. zx sections are shown on the right to show presence of signal inside the vessels. The enrichment of the FISH signals (cumulative area of the spots) in the DA and in the PCV compared to non-endothelial tissues was quantified for each time point (see the “Methods” section for details). Scale bar represents 75 μm. DA, dorsal aorta; PCV, posterior cardinal vein; ISV, intersegmental vessel; PLs, parachordal lymphangioblasts (*n* = 4 embryos (24 hpf); *n* = 5 embryos (32 hpf); *n* = 6 embryos (48 hpf); *n* = 5 embryos (72 hpf); ns = non-significant; ***P < 0.001; **P < *0.01*; *P < *0.05*; Mann–Whitney *U*-test for comparing the expression between the DA and the PCV and Kruskal–Wallis test for testing the endothelial enrichment). **B** Quantification of the expression of *sorbs1* and two veinous/lymphatic markers, *prox1a* and *lyve1b*, in arterial and venous cell types across development from the single-cell sequencing Daniocell database. The color gradient, ranging from light to darker violet, represents the mean gene expression level in cells. The size of each dot corresponds to the percentage of cells expressing the target gene within the cluster

We then checked whether the spatiotemporal expression of *sorbs1* was consistent with the observed phenotypes and could potentially explain their specificity. We performed whole mount fluorescent in situ hybridization (FISH) combined with vessel immunostaining and analyzed the distribution of *sorbs1* transcripts in the main axial vessels of the trunk, the DA, and CV of 24, 32, 48, and 72 hpf zebrafish embryos (Fig. 4A, Additional file 1: Figure S4). We found *sorbs1* signals localizing within ECs of the DA and the CV at each developmental stage, and quantification (Additional file 1: Figure S4, details in the “Methods” section) showed a trend for a higher expression in the vein, with a significant difference at 32 hpf (Fig. 4A). As FISH signals could be difficult to compare across embryos from distinct developmental stages, we mined scRNA-seq data from the Daniocell resource [51]. We analyzed the expression of *sorbs1* in the venous and arterial compartments during development and contrasted it against those of two established markers of lymphatic and venous identity, *prox1a* and *lyve1b* (Fig. 4B). Strikingly, the expression of *sorbs1* was found to be enriched in venous ECs and transiently increased over time, a trend that is very similar to *prox1a* and *lyve1b*, and is compatible with a specific function during lympho-venous sprouting.

### Sorbs1 function in lymphangiogenesis is independent of Vegfc

**Fig. 5:**
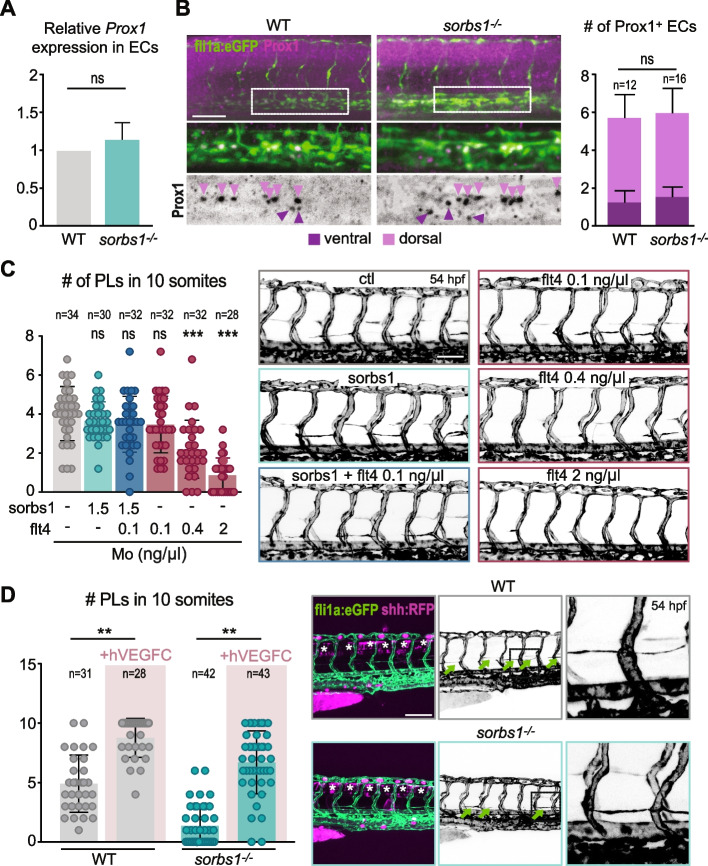
Sorbs1 functions independently of Vegfc signaling during in vivo lymphangiogenesis. **A** RT-qPCR analysis of *prox1a* relative expression at 48 hpf in endothelial cells (ECs) sorted from WT and *sorbs1*^*−/−*^* Tg(fli1a:eGFP)y1* embryos using FACS as described in Fig. 1D (ns = non-significant, unpaired *t*-test). Results are means from 5 experiments. **B** Confocal imaging (Z-maximum intensity projections) of WT or *sorbs1*^*−/−*^* Tg(fli1a:eGFP)y1* embryos after immuno-staining against Prox1 was used to quantify the number of Prox1-positive endothelial cells per 2 body segments in the dorsal (light purple arrows) and ventral side (dark purple arrows) of the posterior cardinal vein at 32 hpf. Scale bar represents 50 μm (*n* = number of embryos; ns = non-significant; two-tailed Mann–Whitney *U*-test). **C** Quantification of PLs in 10 somites of *Tg(kdrl:GFP)* embryos injected with sorbs1 and/or flt4 morpholino, at different concentrations (indicated in the figure) (*n* = number of embryos; ns = non-significant, ***P < 0.001; Mann–Whitney *U*-test). Representative confocal microscopy images (Z-maximum intensity projections) of the trunk vasculature are shown on the right. Scale bar represents 75 μm. **D** Confocal images (Z-maximum intensity projections) were used to quantify PL extent within the trunk region of 54 hpf *Tg(fli1a:eGFP)y1* WT or sorbs1^*−*/*−*^ embryos expressing transgenic constructs coding for human VEGFC fused with RFP, or the RFP alone, under the shh promoter (expression in the roof plate, white asterisks) (*n* = number of embryos; **P < 0.01; Mann–Whitney *U*-test). PLs are indicated with green arrows and magnified insets on the right provide detailed views of PL morphology. Scale bar represents 100 μm

Almost all currently known genetic regulators of zebrafish trunk lymphangiogenesis act through the Vegfc signaling pathway, the major regulator of lymphangiogenesis [53, 54]. Vegfc induces expression of Prox1a in a subset of ECs in the PCV during lymphatic specification, triggering their sprouting, migration, and proliferation to form the lymphatic trunk vessel network. qPCR analysis of *prox1a* expression in ECs showed no significant difference between wild-type and *sorbs1* mutants at 48 hpf (Fig. 5A). In agreement, Prox1 immunostaining on 32 hpf embryos showed similar numbers of Prox1-expressing ECs, including the few ventrally localized ones (Fig. 5B). Live imaging of the *TgBAC(prox1a:KalTA4-4xUAS-ADV.E1b:TagRFP)*^*nim5*^ line confirmed the presence of Prox1a-positive ECs in the PCV of *sorbs1*^*−/−*^ mutants (Additional file 1: Figure S5A). However, Prox1a-positive cells from *sorbs1*^*−/−*^ mutants consistently failed to sprout out of the axial vein, indicating that *sorbs1* is dispensable for lymphatic specification but seems to be required for subsequent migration of LECs (Additional file 1: Figure S5A).

To directly test a link between Sorbs1 and Vegfc signaling we took two orthogonal approaches and analyzed the lymphatic network of double *sorbs1/vegfc* heterozygous or double *sorbs1/flt4* morphant zebrafish embryos as both models have proven suitable to reveal genetic interactions with Vegfc signaling in lymphangiogenesis [33, 55]. We found no evidence of genetic interaction between these two genes (Fig. 5C, Additional file 1: Figure S5B). In line with these findings, forced expression of Vegfc from the floor plate [33] in *sorbs1* mutant embryos increased formation of PLs and TD, indicating that *sorbs1*^*−/−*^ ECs are not affected in their potential to respond to ectopically produced Vegfc (Fig. 5D, Additional file 1: Figure S5C). In agreement with the idea that Sorbs1 functions during lympho-venous sprouting independently of Vegfc signaling, the level of *flt4* expression in the PCV just before sprouting at 30 hpf was unperturbed in *sorbs1*^*−/−*^ mutants (Additional file 1: Figure S5D).

### Regulation of angiogenic structures by Sorbs1 is correlated with BMP signaling

**Fig. 6:**
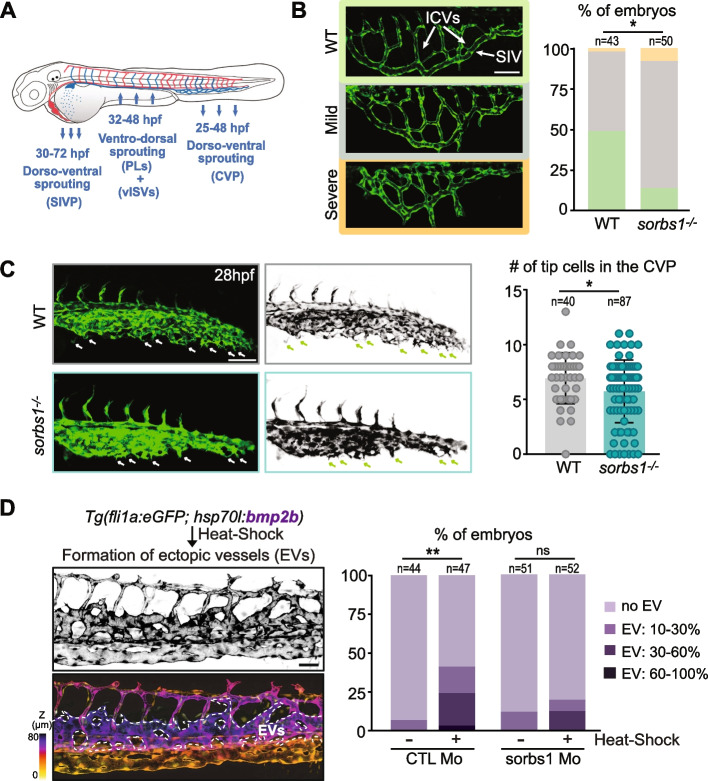
Sorbs1 depletion results in defects in ventral sprouting from the PCV. **A** Schematic representation of arterial (red) and venous (blue) vascular network in the zebrafish embryo. Blue arrows indicate the direction of endothelial cell migration during the formation of PCV-derived angiogenic structures at specific developmental time points. SIVP, subintestinal venous plexus; PLs, parachordal lymphangioblasts; vISVs, venous Intersegmental vessels; CVP, caudal vein plexus. **B** Confocal pictures of the subintestinal plexus of three different phenotypes encountered in WT *and sorbs1*^*−/−*^* Tg(fli1a:eGFP)y1* embryos at 80 hpf and quantification of the distribution of these phenotypes coded according to the color of the box in the two genotypes (*n* = number of embryos, *P < *0.05*; two-tailed Mann–Whitney *U*-test). Scale bars represent 50 μm. ICVs, interconnecting vessels; SIV, subintestinal vein. **C** Confocal imaging (Z-maximum intensity projection) of CVP tip cells (white arrows) from 28 hpf wild-type and *sorbs1*^*−/−*^ embryos used to quantify tip cell numbers (*n* = number of embryos; *P < *0.05*; two-tailed Mann–Whitney *U*-test). Scale bars represent 100 μm. **D** Z-maximum projections, color-coded or not, of confocal images of the trunk regions of 54 hpf *Tg(fli1a:eGFP; hsp70l:bmp2b)* embryos that were heat-shocked at 26 hpf used to illustrate formation of ectopic vessels (EVs, indicated with dotted lines). Scale bars represent 50 μm. Quantification of EVs growing from the PCV at 28 hpf in *Tg(fli1a:eGFP;hsp70l:bmp2b)* embryos injected with Ctl or *sorbs1* Mo without ( −) or after ( +) a heat-shock treatment at 26 hpf (*n* = number of embryos, **P < *0.01*, ns = non-significant, *χ*.^2^ pairwise proportion test with Holm correction)

Along with the angiogenic dorsal sprouts, additional vascular structures are established from the PCV (Fig. 6A). Sprouting angiogenesis in the anterior region of the PCV leads to the formation of the SIVP. Several studies have extensively described the development of the SIVP and demonstrated that it forms from cells originating from the ventral side of the PCV, at around 30 hpf [23]. These ECs collectively engage in a process of ventral migration and give rise at 3 dpf to a left and right basket-like plexus composed of vertical ICVs draining into a SIV (Fig. 6B). Formation of the SIVP was affected in the absence of Sorbs1. *Sorbs1*^*−/−*^ and *sorbs1-*knocked-down (KD) embryos showed abnormal SIVP morphology, with irregular branching and, in severe cases, absence of the surrounding SIV (Fig. 6B and Additional file 1: Figure S6A). Starting at 25 hpf, venous angiogenic sprouts also emerge in the caudal region of the PCV and migrate ventrally through active angiogenesis to form the primordial caudal vein plexus (CVP), a complex network of vessels. During this process, ECs from the caudal vein extend protrusions towards the ventral region of the trunk to migrate and connect with each other to form the CVP at 48 hpf. We observed that while forming, the CVP from *sorbs1* mutant and KD embryos produced fewer ventral sprouts (Fig. 6C, Additional file 1: Figure S6B). In summary, phenotypic characterization of *sorbs1* morphants and mutants revealed phenotypes linked to defects in the development of every major angiogenic structure that originates from the PCV. Interestingly, some of these processes rely on the bone morphogenetic protein (BMP) pathway. More specifically, BMP signaling promotes ventral venous sprouting during CVP development and collective EC migration during SIV ventral expansion [24]. Its role during dorsal secondary sprouting is less clear. To examine the role of Sorbs1 in BMP-induced venous angiogenesis, we used the *Tg(hsp70l:bmp2b)* line, in which ectopic endothelial sprouting can be specifically induced from the PCV by heat-shock treatment (Fig. 6D) [56]. When double transgenic *Tg(hsp70l:bmp2b; fli1a:eGFP)* embryos were heat-shocked at 39 °C for 30 min at 26 hpf (i.e., at the onset of PCV secondary sprouting), 40% showed ectopic vessels (EVs). Sorbs1 KD significantly reduced Bmp-induced sprouting from the PCV, since less than 20% of *sorbs1* morphants displayed EVs after heat-shock. In these embryos, EVs were also visible in a smaller proportion of somite segments, demonstrating that Sorbs1 is implicated in the venous EC response downstream of, or acting in parallel to, BMP.

### Sorbs1 controls EC adhesion through small RhoGTPases

In order to understand the cellular and molecular mechanisms underlying Sorbs1 function during venous sprouting, we generated primary venous ECs deficient for Sorbs1 using small interfering RNA (siRNA) that efficiently and specifically suppresses the expression of Sorbs1, without affecting the viability or proliferation (Additional file 1: Figure S7A, B). In agreement with the observed impairment in EC migration from the PCV in vivo, downregulation of Sorbs1 correlated with a significant decrease in EC migratory capacities in vitro (Additional file 1: Figure S7C).

**Fig. 7:**
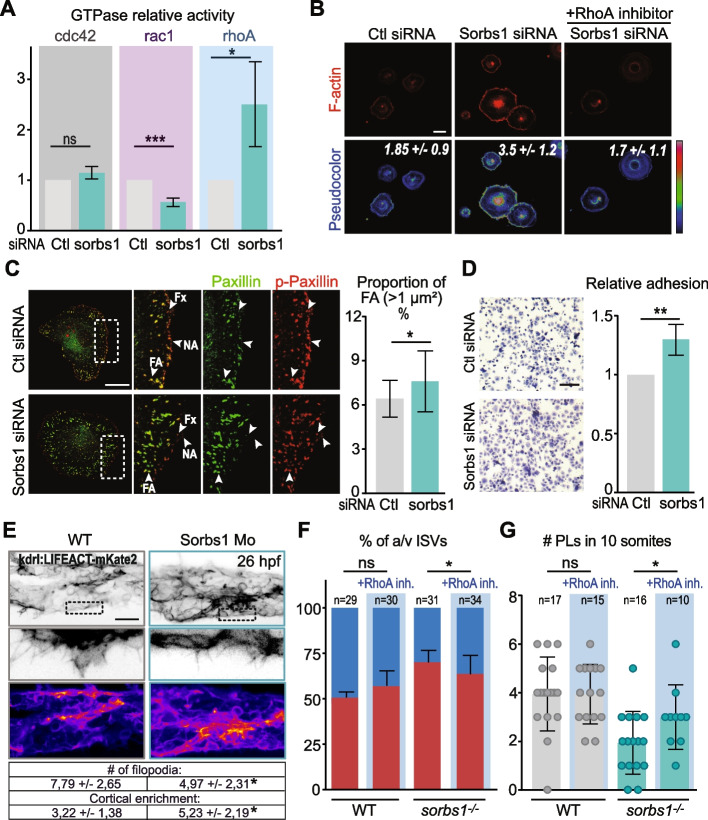
Sorbs1 controls EC adhesive properties via RhoGTPases in vitro and in vivo. **A** Cdc42, Rac1, and RhoA activity in HUVECs transfected with control (Ctl) or *Sorbs1* siRNA. Histogram is from Western blot densitometric analysis of three independent pull-down experiments and represents the ratio between bound active- and total amount of each RhoGTPase in the lysate, relative to control cells (***P < *0.001*, *P < *0.05*, ns = non-significant, Student’s *t*-test). **B** Confocal pictures of peripheral F-Actin (phalloidin staining) in Ctl or *Sorbs1* siRNA transfected HUVECS treated ( +) or not ( −) with the C3 RhoA inhibitor. Images are shown using an intensity-based look-up table (from blue = low to red = high). Numbers represent the average signal intensity ± SD in each condition. Scale bar represents 50 μM. **C** Adhesion complexes were analyzed by confocal microscopy after immunostaining of Paxillin and phospho-Paxillin (p-Paxilin) in HUVECs transfected with control or *Sorbs1* siRNA. Scale bar represents 20 μm. Nascent adhesions (NA) and focal complexes (Fx) are characterized by their small size, peripheral location and high p-Paxillin/Paxillin ratio content (arrowheads). Larger and more mature focal adhesion (FA) were defined as bigger than 1 μm^2^, and their proportion in each condition was quantified (*n* = 21; *P < *0.05*, Student’s *t*-test). **D** Representative micrographs and quantification of adhesion assays performed with HUVECs transfected with control or *Sorbs1* siRNA as described in the “Methods” section. Scale bars represent 100 μm (*n* = 3 independent experiments; **P < *0.01*, Student’s *t*-test). **E** Representative live confocal images (Z-maximum intensity projection) of emerging filopodia in the CVP of 26 hpf *Tg(LIFEACT:mKate2)* embryos injected with Ctl or Sorbs1 morpholino at 5 ng/ul. Enrichment of actin at the cell cortex from the boxed area is visualized using a color look-up table (intensity scale). The number of filopodia per 40 μm and the cortical enrichment of the F-actin signal is indicated for both conditions in the table. Scale bar represents 40 μm (*n* = 7/WT and *n* = 9/Sorb1 Mo, *P < *0.05*, Mann–Whitney *U*-test). **F** WT and *sorbs1*^*−/−*^ embryos were treated or not with RhoA inhibitor at 26 hpf, and the percentage of aISV/vISV at 48 hpf was quantified (*n* = number of embryos; * *P* < *0.05*; ns = non-significant; *χ*^2^ with Yates correction). **G** Quantification of PL number in 10 somites at 54 hpf in WT and *sorbs1*^*−/*^.^*−*^ embryos injected with RhoA inhibitor at 26 hpf or left untreated (*n* = number of embryos; *P < *0.05*; ns = non-significant; Mann–Whitney *U*-test)

Members of the SoHo family are thought to function by interacting with and coordinating the activity of actin cytoskeleton regulators, including RhoGTPases [45, 57–60]. During zebrafish CVP formation, BMP has been shown to affect EC migration by promoting endothelial filopodia extension via activation of Cdc42 [61]. We thus assessed the activity of Cdc42 by performing Rho GTPase activity assays in control and Sorbs1-depleted ECs. Levels of active Cdc42 were similar in control or knocked down cells (Fig. 7A). In contrast, when looking at the other Rho GTPase members, we found that KD of Sorbs1 correlated with a significant up-regulation in RhoA and a marked decrease in Rac1 activities (Fig. 7A). Decreased Rac1 activity was associated with reduced phosphorylation of Rac1 effector kinases PAK2 and PAK4 (Additional file 1: Figure S7D). Activation of RhoA was confirmed by looking at actin polymerization at the lamellipodia of spreading Sorbs1-KD cells, which exhibited a denser network of actin bundles at the cell periphery. Treatment with the C3 Transferase RhoA inhibitor prevented appearance of peripheral F-actin in Sorbs1-KD cells, confirming the causative role of RhoA (Fig. 7B). To get more insight into the cellular function of Sorbs1, we checked its subcellular localization in ECs and found that it localizes at cell-ECM adhesions (Additional file 1: Figure S7E). The formation and maturation of integrin adhesions at the leading edge of migrating cells is controlled by a precise spatiotemporal balance between the activities of Rac1 and RhoA GTPases [62]. Rac1 not only promotes the formation of new adhesions in regions of membrane protrusions but also regulates adhesion turnover through downstream effectors such as PAKs and local inhibition of RhoA [63]. In contrast, RhoA activation is associated with actomyosin-dependent stabilization and maturation of adhesions [62, 64]. We examined the possibility that Sorbs1 might control EC adhesion dynamics by modulating the activity of Rac1 and RhoA. Inactivation of Sorbs1 resulted in alterations in the pattern of EC-ECM adhesions (Fig. 7C). Cell-ECM adhesions found at membrane protrusions are usually divided into two types, depending on their maturation stage. The first adhesions to appear are nascent adhesions (NA) and focal complexes (Fx), which are small dot-like structures characterized by their high content in tyrosine-phosphorylated signaling molecules, such as phospho-Paxillin [65]. Few of them will elongate centripetally and mature into larger (area > 1 μm^2^) focal adhesions (FAs), in a process relying on actin filaments [64]. Compared to control siRNA-treated ECs, Sorbs1 KD cells had a higher proportion of large FAs, which were localized more centripetally (Fig. 7C). In contrast, the proportion of small phospho-Paxillin positive adhesions was reduced at the periphery of Sorbs1-deficient cells (Additional file 1: Figure S7F). In agreement with more mature adhesions, the level of phospho-paxillin was downregulated in sorbs1 KD cells compared to control (Additional file 1: Figure S7G). Importantly, the excessive accumulation of stable FAs was correlated with a significant increase in cell adhesion onto fibronectin, providing a potential explanation for the migration defects in Sorbs1-deficient cells (Fig. 7D).

Cell adhesion onto fibronectin, a process that induced expression of Sorbs1 (Additional file 1: Figure S7H) triggers formation of the FAK-Src complex [66], which is known to induce activation of Rac1 and transient suppression of RhoA, thus promoting adhesion disassembly at cell protrusions. As Sorbs1 localizes to FAs and interacts with FAK, Src, and several of their substrates at ligand-bound integrin adhesions [41, 45, 57, 67], we assessed the activity of this complex upon Sorbs1 depletion. We found that activation upon adhesion of FAK, Src and their downstream target ERK was decreased in Sorbs1-depleted cells (Additional file 1: Figure S7I).

To investigate the impact of Sorbs1 on cytoskeletal rearrangements in vivo, we imaged *Tg(kdrl:LIFEACT-mKate2)* transgenic embryos in which endothelial F-actin is fluorescently labeled and compared actin structures in the actively sprouting CVP of control or *sorbs1* morphant embryos (Fig. 7E). We observed less filopodia emanating from the ventral side of the CVP in *sorbs1* morphants, probably reflecting the decrease in tip cell number (Fig. 6C, Additional file 1: Fig. S6B). In agreement with a role for Sorbs1 in cytoskeleton dynamics during venous sprouting, actin organization appeared different in ECs from *sorbs1* morphants, with a stronger enrichment in cortical actin, a structure that regulates both cell–cell and cell–matrix adhesion complexes (Fig. 7E).

The above data suggest that Sorbs1 participates in the FAK-Src signaling module, which controls the balance between RhoA and Rac1 activities and regulates adhesion dynamics during EC migration. In that case, one should expect that preventing hyperactivation of RhoA would rescue the defects associated with Sorbs1 deficiency. To test this hypothesis in vivo, we treated zebrafish embryos at 26 hpf with the C3 transferase RhoA inhibitor and examined the formation of the vascular structures originating from EC sprouting from the PCV. We used low doses of the C3 RhoA inhibitor, which had no significant impact on the vascular development of wild-type embryos (Fig. 7F, G, Additional file 1: Figure S7J). In contrast, treatment with C3 significantly improved the number of sprouting ECs in the developing CVP and the proportion of vISVs in *sorbs1* mutants (Fig. 7F, Additional file 1: Figure S7J). Similarly, RhoA inhibitor injection improved lymphangiogenesis in *sorbs1*^*−/−*^ embryos, as PL formation was significantly increased (Fig. 7G). In agreement with our hypothesis, these observations demonstrate that the PCV sprouting defects associated with Sorbs1 deficiency are at least in part mediated by RhoA hyperactivation.

## Discussion

Although previous studies have described inactivation of SoHo family members in various animal models [68–71], no vascular phenotype was reported. Here, using the zebrafish model, we provide the first line of in vivo evidence that Sorbs1 is crucial for venous and lymphatic angiogenesis in vertebrates. The endothelial function of Sorbs1 is cell autonomous and appeared to be conserved throughout vertebrates as the defects in the zebrafish vasculature could be rescued by endothelial re-expression of the human ortholog.

The process of trunk secondary sprouting from the PCV is particularly illustrative of changes in cell identity that can occur during vasculogenesis in vertebrates. After their simultaneous budding from the PCV, venous sprouts rapidly diverge and develop into two differently fated structures: the intermediate pool of midline PLs that later give rise to the trunk lymphatic system and the ISVs that establish the venous intersegmental network. Expression of Prox1a in some ECs from the PCV correlates with their lymphatic fate [15, 34]. Yet, recent data suggest that the arterial or venous fate of secondary sprouts could rather be defined upstream of secondary sprouting, at least partially by a Notch-driven heterogeneity preexisting in the primary ISVs [21]. Our data show that lack of Sorbs1 has no obvious effect on Prox1a specification. However, it severely impairs secondary sprouting capacities of ECs associated with both venous and lymphatic fates. Together with a spatiotemporal expression profile that notably fits with establishment of these developmental programs, this would position Sorbs1 as part of the cellular machineries required for the early common morphogenetic events underlying EC secondary sprouting from the PCV.

Migration of the secondary lympho-venous sprouts has been shown to rely on Vegfc signaling [30, 54]. Even though this master lymphangiogenic driver controls the early process of Prox1a induction [16, 26], some Vegfc downstream effectors impact LECs behavior without affecting Prox1a specification [16, 35]. This supports the model that Vegfc might control various steps along the lymphangiogenic process. Our observations suggest that Sorbs1 regulates lymphangiogenesis independently of Vegfc signaling. Genetic interaction experiments demonstrated that Sorbs1 and Vegfc very likely act in distinct pathways. In addition, *sorbs1*^*−*/*−*^ mutant embryos remained highly responsive to ectopic Vegfc induction. Although their number does not reach the wild-type levels, the PLs formed in response to Vegfc in *sorbs1* mutants are morphologically indistinguishable from controls. The fact that the PL defects associated with Sorbs1 deficiency observed under physiological Vegfc levels can be rescued by providing the cells with an excess of the ligand suggests that hyperactivation of Vegc-regulated processes might have the potential to overcome Sorbs1 deficiency. For instance, one could imagine that the observed ERK phosphorylation deficiency is caused by a defective distinct receptor tyrosine kinase that might be partially compensated by Vegfc overactivation. Whereas we also demonstrated that expression of the Vegfc receptor *flt4* is unaffected in the absence of Sorbs1, more experiments would be needed to exclude any involvement of Sorbs1 in the several processes regulated by Vegfc during lymphangiogenesis. The dichotomy between Sorbs1 and Vegfc would be rather unique as other known regulators of PL and TD formation mostly function within the Vegfc pathway.

Examination of other bed specific angiogenic processes gave us additional insights about potential signaling pathways in which Sorbs1 could function during blood and lymphatic vessel formation. Although we did not perform systematic analysis, head vascularization, which displays strong organotypic signatures, appeared unaffected in *sorbs1*^*−/−*^ mutants. Whereas the absence of periorbital edema could be indicative of a lack of defects in facial lymphangiogenesis [72], a more thorough analysis of the lymphatic network in specific transgenic lines would be needed to ascertain the impact of Sorbs1 on facial lymphangiogenesis. In contrast, *sorbs1*^*−/−*^ mutants have alterations of the CVP and SIV networks, two structures originating from ventral migration of ECs out of the axial vein. Initiation of CVP formation [22] and SIVP outgrowth [23, 24] specifically rely on BMP signaling. We observed a clear involvement of Sorbs1 in BMP-induced ectopic sprouting from the CVP. Apart from a morpholino-based study suggesting a role for type II BMP receptors in PL formation [73], the potential involvement of BMP signaling during the early steps of lymphatic network formation has never been precisely characterized beyond LEC specification, survival, and maturation [74–76]. Interestingly, BMP signaling contributes to the endothelial heterogeneity existing in different vessels, its signaling output being dependent on the cellular environmental conditions, such as blood flow and tensions from the ECM [75]. As SoHo proteins are known actors in ECM mechanosensing and mechanotransduction [47], it would be interesting to investigate the connections between Sorbs1, ECM, and BMP signaling in the lymphatic defects observed in *sorbs1* mutant embryos. The context-specific vascular phenotypes of *sorbs1* mutants are highly remarkable and suggest that this cytoskeleton-associated protein participates in establishing EC specificities required throughout PCV-derived secondary venous and lymphatic beds. Lympho-venous sprouting and CVP formation are particularly sensitive to microtubule-associated properties [77, 78]. Disconnection of the leading ECs from the original vessel during SIV formation is not observed during formation of ISV and CVP, suggesting distinct underlying mechanisms [79]. Using cell culture experiments, we showed that *Sorbs1-KD* ECs display altered adhesion dynamics and migration. Interestingly, the CVP phenotype in *sorbs1* mutants is strikingly similar to that of zebrafish embryos lacking various components of the ECM such as fibrillins [80, 81], a protein that can interact with different BMPs [82]. Additionally, zebrafish mutant for Polydom/svep1, a large protein involved in cell adhesion to the ECM, failed to form PL and TD lymphatic structures due to sprouting impairment of properly specified LECs [38, 83]. This raises the intriguing possibility that venous plexus and lymphatic network formation might be particularly sensitive to alterations of integrin-mediated cell-ECM adhesions.

Our study not only provides an important cellular and developmental in vivo context for cytoskeleton regulation by Sorbs1, but it also discloses underlying mechanistic aspects. Indeed, we demonstrate that Sorbs1 acts upstream of RhoGTPases to control EC actomyosin cytoskeleton and migratory behavior. Prior to this work, only few studies had alluded to potential connections between SoHo proteins and RhoGTPases signaling [59, 60, 84, 85]. Although extension of filopodia and migration of leading ECs during BMP-induced CVP morphogenesis was shown to be dependent on the Cdc42 RhoGTPase [61], we found that Cdc42 activity was not affected in the absence of Sorbs1 in ECs. While we cannot exclude differences between cultured human venous ECs and zebrafish veins, our results tend to show that Sorbs1 controls the RhoA-Rac1 balance, through the FAK-Src pathway. Integrin-mediated activation of the FAK-Src complex during cell spreading and migration stimulates Rac1 activity and maturation of focal complexes into stable adhesions [86]. Consistent with the idea that it participates in FAK-Src activation, Sorbs1 protein levels are highly and transiently induced following integrin engagement onto fibronectin. FAK and Src also control phosphorylation of p190RhoGAP and in addition to Rac1 activation, Sorbs1 might affect focal adhesion turnover through repression of RhoA activity [87, 88]. Together with the well-described antagonistic regulation of Rac1 and RhoA, these findings would be consistent with the reciprocal increase in RhoA and decrease in Rac1 activities that we observed in Sorbs1-depleted ECs. Based on our observations, it is tempting to speculate that Sorbs1 might contribute to the spatial coordination of RhoA and Rac1 activities within migrating ECs during vascular network expansion. In the absence of Sorbs1, local increase in RhoA and decrease in Rac1 activities would be expected to reduce lamellipodia dynamics and membrane protrusive activity, thus impairing EC migration and therefore vascular integrity. Indeed, it has been reported that the increase of RhoA activity in ECs results in defects in ISV sprouting and vascular integrity [89]. Importantly, we show that defects in PCV secondary dorsal and ventral sprouting associated with Sorbs1 knock-out can be partially rescued by a RhoA inhibitor, indicating that RhoA activation is causal in the vascular phenotype of *sorbs1*^*−/−*^ embryos and suggesting that Sorbs1 affects common EC properties during these processes. How RhoA regulation by Sorbs1 is integrated in the signaling pathways governing these processes is still an open question, but it is worth noting that while ERK activation has been described to participate in LEC migration [54] and CVP formation [22], adhesion-triggered phosphorylation of ERK is reduced in *Sorbs1-KD* ECs.

## Conclusions

In summary, our results show that the Sorbs1 protein participates in key molecular pathways driving stage- or context-specific regulation of EC morphogenic properties during venous and lymphatic vascular development. More specifically, we identify Sorbs1 as a novel genetic regulator of developmental lymphangiogenesis that functions independently of Vegfc signaling. Better understanding of these pathways and identification of novel actors will provide new opportunities that can be exploited for vascular normalization strategies in various diseases.

## Methods

### Zebrafish

Adult fish and embryos were raised under standard conditions according to national ethical and animal welfare regulations as reported in the FELASA guidelines [90]. All animal experiments were approved by the animal welfare committee of the University of Liège (protocol number 14–1556, laboratory agreement number LA 1610002) and the Université libre de Bruxelles (ULB) (protocol number 07GosIBMM, laboratory agreement number LA 1610002). The zebrafish lines used in this study were as follows: *Tg(fli1a:eGFP)*^*y1*^ [91], *Tg(hsp70I:bmp2b)* [92], *TgBAC(prox1a:KalTA4-4xUAS-ADV.E1b:TagRFP)*^*nim*^ [76, 93], vegfc^hu5055^ [33], and Tg(kdrl:LIFEACT-mKate2)^ulb32^ (this study).

### Generation of knock-out lines using CRISPR/Cas9 system

Cas9 mRNA and guide RNAs (gRNAs) were synthesized as described in Jao et al. [94]. Briefly, the Cas9 mRNA was synthesized by in vitro transcription using the T3 mMESSAGEmMACHINE Kit (#AM1348, Ambion). The primers for the generation of DNA templates of gRNAs were designed through the CHOPCHOP software, and a T7 promoter sequence was added to the 5′-upstream of the gRNA sequence. The gRNA was digested by BamHI and then submitted to in vitro transcription using MEGAshortscriptT7 kit (#AM1354, Ambion). The size and quality of the capped mRNA and gRNA were confirmed by electrophoresis through a 2% (w/v) agarose gel. After this, 300 ng/μl of Cas9 mRNA and 100 ng/μl of gRNA were co-injected into one cell-stage zebrafish embryos. Embryos were derived from the transgenic line *Tg(fli1a:eGFP)y1* cross. The injected embryos were raised to adulthood. To test mutagenesis efficiency, we genotyped the zebrafish by extracting the DNA from their fin (FIN-CLIP), followed by PCR and heteroduplex melting annealing (HMA) gel. F0 fish were crossed with *Tg(fli1a:eGFP)y1* fish to generate heterozygous F1 progeny, which were then genotyped by HMA gel and DNA sequencing. Heterozygous F1 zebrafish were crossed with the aim to generate homozygous mutant fish sorbs1^*−*/*−*^ (sorbs1^ulb29^).

### Morpholino, RNA and DNA injection

One-cell stage *Tg(fli1a:eGFP)y1* embryos were injected with 5 ng of Sorbs1 splice-blocking (5′-TCCCCAAATGCTCTTCTTACCAGTA-3′), Sorbs1 ATG-blocking (5′- AGGTCAGGAGAA CTCTTCATGATCC-3′), and control morpholino (5′- CCTCTTACCTCAGTTACAATTTATA-3′). For testing genetic interaction with *flt4*, we used ATG-blocking morpholino against flt4 (5′-CTCTTCATTTCCAGGTTTCAAGTCC-3′) at concentrations indicated in the figure. We performed rescue experiments by injecting RNA molecules (60 ng/μl) from in vitro transcription reactions using linearized PCS2 + vector coding for human Sorbs1. Alternatively, transient mosaic endothelial over-expression was obtained by co-injection of pT2 DNA plasmids and 10 pg of Tol2 transposase mRNA. We injected 15 pg of DNA plasmids containing fli1a-hSORBS1-P2A-NLS-eBFP, 7.5 pg of kdrl-hSORBS1-P2A-NLS-eBFP, or 10 pg of shh-hVEGFC-IRES-RFP^33^. As endothelial promoter, we used the 2062-bp sequence that starts 1079 bp upstream of the ATG (*fli1a*) and the 6449-bp sequence that starts 72 bp upstream of the ATG (*kdrl*). The signal of the fluorescent transgene expression marker was checked in order to take only into account the embryos displaying proper transgene expression.

### RNA extraction and PCR amplification

RNA was extracted from zebrafish embryos using Trizol reagent (Invitrogen) according to the manufacturer’s protocol. RNA from HUVECs was prepared using the nucleospin RNA kit (Macherey Nagel). RNA integrity and concentration were assessed by spectrophotometry analysis (Nanodrop, Thermo Scientific). Reverse transcription reactions were done using the RevertAid H Minus First Strand cDNA Synthesis Kit (Fermentas) with random hexamer primers. The cDNA was then submitted to quantitative real time PCR using Sybrgreen technology (Eurogentec) on a Stepone apparatus (Applied Biosystems) or to end-point PCR amplification followed by gel electrophoresis analysis.

Primers used for end-point PCR are as follows: Zebrafish *sorbs1*: ATCATCGATGTGCACTAACGTG (Forward) and CTCCAGCAGAGGGCACAG (Reverse). Primers used for quantitative real-time PCR are as follows: Zebrafish sorbs1: GCCAGGAAAGTCTTCAGTGC (Forward) and TCTGCTTCACCGTCACTCAC (Reverse); Zebrafish *prox1a*: TGTCATTTGCGCTCGCGCTG (Forward) and ACCGCAACCCGAAGACAGTG (Reverse). Zebrafish *elfa*: CTTCTCAGGCTGACTGTGC (Forward) and CCGCTAGCATTACCCTCC (Reverse). Primers used for mutagenesis efficiency analysis by PCR are as follows: TGAGACTCCAGCAGACATGG (Forward) and ACAATTACAGCTGGAGAACTACA (Reverse).

### Whole mount chromogenic in situ hybridization

An antisense RNA DIG-probe was generated by transcription from linearized pCS1 vector containing Sorbs1 coding sequence using SP6 RNA polymerase kit where UTPs were labeled with digoxigenin (DIG) (Roche, 11,175,025,910). Primers used for amplification of the coding sequence are as follows: GTTCTCCTGACCTCATGCCT (Forward) and GTCCTTCGAGAGCTCAGTGT (Reverse). Whole mount in situ hybridization was performed in 12, 24, and 48 hpf embryos and in 3 dpf larvae. Every time point was fixed with paraformaldehyde 4% overnight at 4 °C and then dehydrated and rehydrated through methanol and PBS 1x (Gibco)-Tween 5% washes. Embryos were permeabilized with 10 μg/mL of proteinase K and then re-fixed with paraformaldehyde 4%. Antisense probe hybridization was performed using 100 ng of sorbs1-DIG-probes hybridization buffer containing 5% dextran sulfate at 65 °C overnight. The use of a DIG alkaline phosphatase-conjugated antibody (Roche, 11,093,274,910, dilution 1/3000), and its substrates BCIP and NBT, enabled the colorimetric detection of sorbs1 transcript. Pictures were taken with an Olympus SZxX10 stereomicroscope.

### Whole mount fluorescent in situ hybridization (FISH) and immunofluorescence

#### Staining

FISH was performed using the same probe as the one for chromogenic in situ hybridization. Every time point was fixed with paraformaldehyde 4% overnight at 4 °C, dehydrated in 100% methanol and then rehydrated through successive incubations in 75%, 50%, and 25% methanol, and then washed in PBS 1x (Gibco)-Tween 5%. Embryos were permeabilized with 20 μg/mL or 40 μg/mL (for 72 hpf embryos) of proteinase K and then re-fixed 20 min with paraformaldehyde 4%. Incubation with an hybridization buffer (50% deionized formamide, 5X SSC, 0.1% Tween-20, pH 5.5) was done for 2 h at 68 °C before adding 200 ng of sorbs1-DIG-probe overnight at 68 °C. Embryos were washed twice in the hybridization buffer, in 2X SSCT, and in 0.2X SSCT and incubated in FISH blocking solution (2% Roche blocking reagent Roche 10,057,177,103 in MABT) for 3 h at room temperature before adding an anti-DIG POD (Merck 11,207,733,910, 1/1000) and an anti-GFP (Aves Labs GFP-1020, 1/200) antibody for an overnight incubation at 4 °C without agitation. Embryos were then washed twice in MABT, and the probe was revealed using TSA Plus Cy3 detection kit (Akoya Biosciences, NEL744001KT, 1/50 for 1 h). After three washes in PBST, embryos were incubated in blocking solution (5% normal goat serum in PBST) for 1 h at room temperature before adding alexafluor488 conjugated secondary antibody (1/500) overnight at 4 °C. Embryos were then washed in PBST, mounted on glass slides in DAKO medium after manually removing their yolk and cutting their head, and then imaged using a Zeiss LSM710/AxioObserver Z1 with a 63X/1.4 oil objective.

#### Analysis

A CellProfiler pipeline was devised to segment the spots of FISH images using the “A trous” wavelet decomposition method, in manually outlined regions of interest. Parts of the DA and the PCV that were properly aligned with the xy axis were manually annotated on the maximum intensity projections in FIJI, and the regions of interest (ROI) were saved via the ROI manager. A custom CellProfiler plugin (loadmaskobjects.py) was used to load these annotations and treat them as mask objects so that spots within the annotations would be recognized as such. Another custom CellProfiler plugin (atrousfilter.py) was devised to apply an “A trous” wavelet decomposition to an image and to extract a set scale corresponding to the spot size. Wavelet transforms can filter round objects within a size bracket and are well suited for spot detection. The output image was then segmented by the “primary objects identification” step. A global threshold strategy was applied, with no filtering and declumping based on intensity. Measurements of interest such as annotation area, number of spots within the annotation, and their cumulative area were then output to a CSV file for final quantification. The cumulative area of the FISH spots within each ROI from the DA or the PCV divided by the total area of the ROI was normalized to the cumulative area of the FISH spots found outside of the DA and the PCV, to give an endothelial enrichment index.

### Phenotyping

Embryos were anesthetized with tricaine 0.4% to perform phenotypical analysis. Experiments from Figs. 2A, B, D, 3A, C, and 5B and Supplementary Figures S1D, S3A, S3B, S5B, and S5C were performed in a blind way (genotyping after phenotyping). Analysis and pictures of overall zebrafish morphology and edemas were performed under a stereomicroscope or using phase contrast imaging on an AxioObserver Z1 microscope using an EC Plan-Neofluar 10x/0.30 objective. Analyses of zebrafish vasculature were performed under a fluorescent stereomicroscope, whereas confocal pictures were taken on live embryos embedded in low melting point agarose (0.8%) on a confocal Nikon A1R or Zeiss LSM710. 3D color projections were done using the volume view-slices mode and the volume view-z depth blending functions of the NIS-Element A1R1 Software. Lightsheet Zeiss Z1 was used in order to perform time-lapse video of the emerging secondary sprouts at 36 hpf from the trunk vasculature of *TgBAC(prox1a:KalTA4-4xUAS-ADV.E1b:TagRFP)*^*nim5*^ zebrafish embryos which were embedded in low melting point agarose (0.8%) with tricaine 0.4%.

For rescue experiments with transgenic overexpression, only embryos with proper expression of the transgene, as observed with the fluorescent signal, were quantified.

For rescue experiments with RhoA inhibitor, control, sorbs1 mutants, or morphants embryos were incubated with C3 Transferase RhoA inhibitor (#CT04-A, C3ytoskeleton, Inc.) (1 μg/mL) at 26 hpf before analyses of CVP structures at 28 hpf and of the proportion of aISV/vISV at 48 hpf. For PL development rescue, RhoA inhibitor (1 μM) was injected in the circulation of wild-type and *sorbs1*^*−/−*^ at 28 hpf, and PLs were quantified with a fluorescent stereomicroscope at 54 hpf.

For testing the interaction with Vegfc, embryos from *sorbs1*^+*/−*^ and *vegfc*^*−/−*^ crosses were submitted to phenotyping before being genotyped. The verification of the *vegfc* allele was performed by DNA amplification (forward primer: ACTGAAAGGAAATAACTTTTG, reverse primer: GTCCAGTCTTCCCCAGTATGT) followed by BsaI digestion, which results in two DNA bands in KO and one band in WT. Assessment of Sorbs1 knock-out was performed by high-resolution melting assay (forward primer: GTGGCCTGTATCAAGGTCGT, reverse primer: TCGACATCAGCATCTTGAGC).

### Live imaging of secondary sprouting

*Tg(fli1a:eGFP)* zebrafish were grown at 28 °C in PTU-containing medium until 30 hpf. They were then immobilized in 1% low-melting point agarose gel containing tricaine in IBIDI chambers and imaged at 28 °C using a Zeiss LSM710/AxioObserver Z1 with a Plan-Apochromat 20x/0.8 M27 objective. Z-stacks were acquired across the sample (every 3 μm) every 20 min for up to 20 h. For quantifying the time spend by the venous sprout before connecting the aISV, we counted the time frames between the first appearance of a cell protrusion from the PCV that is longer than 15 μm and its first contact with the neighboring aISV.

### Live imaging of actin cytoskeleton

*Tg(kdrl:LIFEACT-mKate2)* zebrafish were grown at 28 °C in PTU-containing medium until 26 hpf. They were then immobilized in 1% low-melting point agarose gel containing tricaine in IBIDI chambers and imaged at 28 °C using a Zeiss LSM710/AxioObserver Z1 with a Plan-Apochromat 20x/0.8 M27 objective. Filopodia (longer than 5 μm) emanating from the ventral side of the CVP were manually counted, and the ratio between the signal intensity in the middle of the cell and at the cortex was calculated.

### Prox1 immunostaining

Embryos were fixed at 32 hpf with PFA4% O/N at 4 °C and then washed 4 times 5 min with PBS-T (Tween20 0.5%) before permeabilization using 0.5% TX100 for 1 h at RT. After three PBS-T washes, embryos were put in a blocking solution containing 10% normal goat serum, 1% BSA, 0.2% TX100, and 0.1 M glycine for 2 h at RT. The primary antibody against prox1 (Abcam ab209849) was added in blocking solution at 1:500 dilution and incubated for 3 days at 4 °C under agitation. After extended washes, embryos were incubated with fluorescent secondary antibody (goat anti-rabbit-AF594 from Invitrogen) at 1:5000 in blocking solution O/N at 4 °C under agitation. Embryos were then washed for several hours with PBS-T and kept in PBS before being mounted in 1% agarose gel and imaged with a LSM710/AxioObserver Z1 (Zeiss) with a Plan-Apochromat 20x/0.8 M27 objective. Stacks were acquired every 2.4 μm across the sample depth.

### Fluorescence-activated cell sorting (FACS)

A batch of 200 zebrafish embryos at different developmental time points was euthanized using an overdose of tricaine and transferred to a 35-mm culture dish. The tricaine was removed as much as possible and 1 ml of dissociation solution (HBSS (Ca2 + and Mg2 + Free) + 1% BSA + 20 mM HEPES) was added. During an incubation period of 30 min, the larvae dissociation was advanced by pipetting up and down with a 1-ml pipette every 10 min. When the embryos were completely dissociated, the cells were then washed with PBS and resuspended in 2 ml of resuspension solution (PBS + FCS 10%). The cell solution was passed several times through a 40-μM mesh filter prior to cell sorting. This cell suspension was analyzed by flow cytometry on a Fusion Aria sorter. GFP positive and negative cells were sorted and collected into the lysis buffer of the RNA isolation kit. The cells were kept on ice during the whole procedure.

### Single-cell sequencing analysis

Expression data from the Daniocell database [51] was downloaded, which is a single-cell RNA-seq dataset of whole-animal wild-type zebrafish embryos and larvae across multiple stages of development. Dimensionality reduction was performed to get UMAP plots, on which the normalized expression could be visually displayed. For more in-depth analysis of the vasculature cells, this dataset was subsetted and subsequently assigned either the “arterial” (hema.7, hema.18, and hema.31) or “venous” (hema.2, hema.30, and hema.37) label. The expression of the genes of interest could then be plotted for these two subgroups and over the different developmental stages. We opted to visualize this in a dotplot which shows both the percentage of cells in which the gene is expressed and the average expression value in these cells.

### Cell culture and transfection

Human umbilical vein endothelial cells (HUVECs), human dermal microvascular endothelial cells (HDMECs), human mammary epithelial cells (HMECs), human umbilical artery endothelial cells (HUAEC), human embryonic kidney 293 cells (HEK 293), human dermal lymphatic microvascular endothelial cells (HMVEC-dLyAd), and HeLa cells were obtained from Lonza. All functional assays were performed with HUVECS which were grown at 37 °C in endothelial basal medium (EBM) supplemented with hydrocortisone (1 μg/ml), bovine brain extract (12 μg/ml), gentamicin (50 μg/ml), amphotericin B (50 ng/ml), epidermal growth factor (10 ng/ml) (Lonza), and 10% fetal bovine serum (FBS, Perbio). Transfections of siRNA were performed using the GeneTrans II (MoBiTec) reagent according to the manufacturer’s protocols.

Except for scratch-wound assays, all functional assays were performed on fibronectin-plated HUVECs. Briefly, cells were harvested, left 30 min in suspension to recover from trypsinization, and seeded onto fibronectin-coated dishes for 30 min.

### Antibodies and RNA interference (RNAi)

Anti-Sorbs1 was obtained from Abcam (#Ab4551). Anti-PAK4 (#3242), PAK2 (#2608), Src (#2123), ERK1/2 (#9102), and phosphorylated Src (#2101S), paxillin (#2541S), and ERK1/2 (#9101) were purchased from Cell signaling. Anti-paxillin (610,051), FAK (#ab72140), and its phosphorylated form (# 44-624G) were from Biosciences, Abcam, and Invitrogen, respectively. Non-targeting control siRNA and siRNA duplexes targeting Sorbs1 (5′-UUAAGUCCUGAGUGCUCUUC-3′) were synthesized and purchased from Eurogentec.

### Migration assays

Migration assays were performed as described [60]. Briefly, for the scratch assay a confluent HUVEC monolayer was wounded 48 h after siRNA transfection, using a sterile P200 tip to create a cell-free zone. For each wound, two different fields were photographed just after injury (*t* = 0 h) and 16 h later. Quantification of cell migration was made by measuring the percentage of area recovery using the ImageJ software in 12 fields from 3 independent experiments.

### Adhesion assay

Adhesion assays were performed essentially as described with slight modifications [60]. Forty-eight hours after transfection, HUVECs were seeded on fibronectin precoated-wells for 30 min. After extensive washing with PBS, remaining cells were stained with crystal violet. The dye was released by cell permeabilization and directly proportional to the number of cells; dye concentration was measured by reading absorbance at 560 nm.

### Proliferation assays

Forty-eight hours post transfection with siRNA, semi-automatic cell counting assessment of proliferation was performed. Briefly, 24 h post-transfection with siRNA, cells were seeded at 20,000 cells/well in 24-well plates in triplicate. Cells were counted using the Scepter 2.0 Handheld Automated Cell Counter (Millipore) over a 2-day period and proliferation curves were generated by plotting the average cell number over time.

### Immunohistochemistry

AccuMax Array (A301 VI) slides were stained with goat anti–human Sorbs1 (Abcam, ab4551) antibody. Slides were incubated overnight in optimized dilutions of primary antibodies in Antibody Diluent (Dako, S2022). Peroxidase-conjugated anti-goat Ig (Vector) was then added for 1 h. Revelation was performed using diamino-3, 3′ benzidine (DAB) according to standard protocols. Images were acquired by using a FSX100 microscope (Olympus).

### Immunofluorescence

For immunofluorescence experiments, HUVECs were seeded onto fibronectin coated coverslips 48 h after siRNA transfection. Cells were fixed after 30 min in 4% paraformaldehyde, permeabilized with PBS-TritonX 0.1%, and incubated overnight with the appropriate primary antibody dilutions in PBS-BSA 4%. Cells were then incubated with appropriate secondary antibody dilutions for 1 h. After washing, cells were mounted with Mowiol (Sigma) and processed for immunofluorescence using a confocal Nikon A1R.

### Rho GTPase pull down activity assay

SiRNA-treated HUVECs were cultivated for 30 min on fibronectin. After harvesting, total cellular active RhoA levels were measured using the Rho Activity Assay (Cytoskeleton Inc., BK036) following the manufacturer’s guidelines. In short, cell lysate (approximately 500 μg of total protein) was incubated for 1 h at 4 °C with GST-Rhotekin beads. Bound activated RhoA was eluted from the beads and analyzed by western blotting using a RhoA antibody. The levels of active Rac1 and Cdc42 were measured using the Rac1 and CDC42 Activity Assay (Cytoskeleton Inc., BK035 and BK034, respectively); cells were lysed in a buffer containing 5 mM DTT, 50 mM Tris pH 7.2, 1% tritonx-100 (10%), 0.5% deoxycholate (20%), 0.1% SDS (20%), and 500 mM NaCl 5 M. Extracts were then incubated 1 h at 4 °C with GST-PAK beads. Bound activated Rac1 and Cdc42 were eluted from the beads and analyzed by western blotting using dedicated antibodies.

### Statistical analysis

Unless stated otherwise, experiments were performed at least three times independently and graphs represent means ± standard deviation. Normality tests were performed, and when the data were considered normal, statistical analysis were performed by two tailed Student’s *t*-test or a Pearson’s chi-squared test. Mann–Whitney *U* and Wilcoxon rank-sum test were used otherwise.

### Supplementary Information

**Additional file 1:Supplemental Figure 1.** Characterization of Sorbs1 depletion in vivo and its expression in endothelial cells. (A) Phylogenetic tree was constructed from human, mouse and potential zebrafish mRNA of *Sorbs1*, *Sorbs2* and *Sorbs3* using ClustalW software. One zebrafish ortholog was identified for *sorbs1* (*Dr sorbs1*) and *sorbs3* (*Dr sorbs3*) and two for *sorbs2* (*Dr sorbs2a* and *Dr sorbs2b*). (B) DNA and amino-acid sequences of the region around the CRISPR/Cas9 target site of the wild-type sorbs1 allele (WT) and its 14-bp deletion (-14) following CRISPR/ Cas9-based editing. (C) Western blotting analysis of protein extracts from wild-type (WT) and Sorbs1 mutant (*sorbs1*^*−/−*^) embryos, using anti-Sorbs1 antibody. GAPDH was used as loading control. (D) Phase-contrast imaging of 48 hpf embryos (upper panel) and zooms on the somites from trunk regions (down panel) of wild-type (WT) and *sorbs1*^*−/−*^* Tg(fli1a:eGFP)* embryos used to quantify the antero-posterior (AP) body length (white line), the brain size (dashed white line) and the somite angle (white circular arc) and AP length (white line) (*n* = number of embryos, ns = non-significant, Mann–Whitney U-test). Scale bars represent 500 μm. (E) Expression of Sorbs1 in various human tissues assessed by immunohistochemistry. Typical Sorbs1 staining in endothelial cells is illustrated for the indicated tissues. Boxes correspond to the enlarged area showing expression of Sorbs1 in blood vessels (arrows). Scale bars represent 50 μm and 500 μm respectively in large and zoomed picture. (F) Western blotting analysis of Sorbs1 expression in various human endothelial cells: HDMECs (Human Dermal Microvascular Endothelial Cells), HMECs (Human Mammary Epithelial Cells), HUAECs (Human Umbilical Artery Endothelial Cells), HUVECs (Human Umbilical Endothelial Cells), HMVEC-dLyAd (Human Dermal Lymphatic Microvascular Endothelial Cells), HEK293 (Human Embryonic Kidney 293) and Hela cells. HSP90 was used as a loading control. (G) Whole mount in situ hybridization using a digoxigenin-labeled antisense sorbs1 probe at the different indicated time points. Scale bars represent 100 μm. **Supplemental Figure 2.** Sorbs1 knockdown recapitulates *sorbs1*^*−/−*^ phenotypes. (A) Maximal intensity projection of a confocal z-stack of the cranial vasculature of *Tg(fli1a:eGFP)y1* wild-type (WT) and *sorbs1* mutant (*sorbs1*^*−/−*^) embryos at 60 hpf in dorsal views (anterior to the left) and wire diagram of the brain vasculature in dorso-lateral view. Red vessels in the 3D renderings represent the intra-cerebral central arteries (CtAs) and gray vessels represent the perineural vessels (primordial hindbrain channels: PHBC and basilar artery: BA). Quantification of the corresponding hindbrain CtAs in wild-type (WT) and *sorbs1* homozygous (*sorbs1*^*−*/*−*^) embryos at 60 hpf. Error bars represent median ± interquartile range; (*n* = number of embryos; ns = non-significant, Kruskal–Wallis test). Scale bar represents 100 μm. (B) Analysis of *sorbs1*^*−/−*^ embryo survival over time (4–10 dpf) in relation to their TD defects. TD defects were classified based on the percentage of its development through 10 somites (total number of analyzed embryos: 70). (C) RT-PCR analysis on total RNA from embryos injected with 5 pg control morpholino (Ctl Mo) or with a splice-blocking morpholino against *sorbs1* (sorbs1 Mo). (D) Western blotting analysis of total protein extracts from 48 hpf embryos injected with control (Ctl), ATG- and splice-blocking morpholino against *sorbs1*, using an anti-Sorbs1 antibody. GAPDH was used as loading control. (E) Quantification of PLs was performed at 48 hpf between 10 somites in Ctl and *sorbs1* morphant embryos (*n* = number of embryos; ****P < 0.001, ***P < 0.001, *P < *0.05*; two-tailed Mann–Whitney *U*-test). Decreasing doses of two types of morpholino were injected at one cell stage, one that blocks sorbs1 splicing (splic.) and one that blocks mRNA translation (ATG). (F) Quantification of thoracic duct (TD) formation was performed at 4 and 6 dpf between 10 somites in embryos injected with Ctl or decreasing doses of the *sorbs1* splice-blocking morpholino (*n* = number of embryos; ****P < *0.001*, ***P < 0.001, *P < *0.05*; ns = non-significant, two-tailed Mann–Whitney *U*-test). (G) Z-maximum projections of confocal images of the trunk vasculature of 54 hpf *Tg(fli1a:eGFP)y1* embryos injected with ctl or sorbs1 morpholino and expressing transgenic endothelial constructs coding for human Sorbs1 or not were used to quantify the number of PLs in 10 somites. BFP is used as transgenesis marker. Scale bar represents 50 μm (*n* = number of embryos; **P < 0.01, *P < *0.05*; Mann–Whitney *U*-test). **Supplemental Figure 3.***Sorbs1* depletion impairs secondary sprouting from the PCV. (A) Frames (Z-maximum projections) from time-lapse confocal imaging of *sorbs1*^*−/−*^* Tg(fli1a:eGFP)y1* embryos during venous secondary sprouting. Scale bar represents 50 μm. Numbered black arrows follow sprouting events from the PCV, one of them being illustrated with zooms spanning the time indicated with the dashed arrows in the last boxes. (B) Quantification of the time (in minutes) between the emergence of a venous sprout to its connection to an pre-existent aISV in WT and *sorbs1*^*−/−*^ Tg(fli1a:GFP) embryos imaged as described in A. *n* = number of embryos; *P < 0.05; two-tailed Mann–Whitney *U*-test). (C) Quantification of secondary sprouts in control and *sorbs1*-morphant embryos (splice-blocking morpholino) at 36 hpf was established using the five indicated categories (*n* = number of embryos; **P < 0.1; two-tailed Mann–Whitney *U*-test). (D) Percentages of vISVs and aISVs were quantified at 48 hpf in a 10 somite region in the trunk of embryos injected with control or *sorbs1* splice-blocking morpholino (*n* = number of embryos; ***P < 0.001; χ^2^ without Yates correction). **Supplemental Figure 4.***Sorbs1 expression in zebrafish embryos*. Illustration of the methodology used to quantify Sorbs1 FISH signals in Figure 4A. Confocal images of sorbs1 expression (magenta/white dots) and of the vessels (green) of *Tg(fli1a:GFP)y1* embryos were used to quantify the distribution of sorbs1 transcripts in the DA and the PCV. The FISH signal was filtered using a wavelet decomposition method, the spots were detected and their cumulative area was quantified in the DA and the PCV. **Supplemental Figure 5.** Sorbs1 functions independently of Vegfc signaling during in vivo lymphangiogenesis. (A) Frames (Z-maximum projections) from time-lapse lightsheet imaging of Prox1 expressing ECs sprouting from the posterior cardinal vein in WT and sorbs1^*−*/*−*^ TgBAC(prox1a:KalTA4-4xUAS-ADV.E1b:TagRFP)^nim5^ embryos (the green cell in the scheme on the left). Scale bars represent 25 μm. ISVs: intersegmental vessels; DA: dorsal aorta; PCV: posterior cardinal vein. (B) Quantification of PL extent within the trunk region of 54 hpf Tg(fli1a:eGFP)y1 embryos from the indicated genotype resulting from the incross of sorbs1^+/*−*^ with vegfc^+/*−*^ embryos (*n* = number of embryos; ns = non-significant). (C) Quantification of the trunk TD extent (4dpf) in WT or *sorbs1*^*−/−*^* Tg(fli1a:eGFP)y1* embryos expressing or not a transgenic constructs coding for human VEGFC under the shh promoter (*n* = number of embryos; **P < 0.01; Mann–Whitney *U*-test). (D) Confocal images of FISH staining used to determine flt4 expression (magenta) in WT and *sorbs1*^*−/−*^* Tg(fli1a:eGFP)y1* embryos at 30 hpf. Quantification of the fluorescent flt4 signal intensity was performed in the PCV (*n* = 5 embryos; ns = non-significant; Mann–Whitney *U*-test). Scale bar represents 50 μm. DA: dorsal aorta; PCV: posterior cardinal vein. **Supplemental Figure 6.***Sorbs1* depletion leads to defects in ventral sprouting from the PCV. (A) Quantification of subintestinal plexus phenotypes in embryos injected with control or *sorbs1* Mo (*n* = number of embryos; ***P < 0.001; two-tailed Mann–Whitney *U*-test). (B) Quantification of tip cell numbers were performed at 28 hpf in embryos injected with control or *sorbs1* Mo (*n* = number of embryos; ***P < 0.001; two-tailed Mann–Whitney *U*-test). **Supplemental Figure 7.** Sorbs1 deletion affects the migratory and adhesive properties of endothelial cells. (A) HUVECs were transfected with control or *Sorbs1*-targeting siRNA. Efficiency of RNA silencing was assessed by Western blotting analysis using Sorbs1 specific antibody 48 h after transfection. Actin was used as loading control. (B) HUVECs were transfected as in (A), harvested 24 h after transfection and plated in triplicate at a defined density. Cell number was then assessed by semi-automatic counting at 48 h and 72 h after transfection. Results are presented as the average ± SD increase in cell number, from 3 independent experiments (ns = non-significant, Student’s t test). (C) Micrographs representing HUVECs transfected with control siRNA or with siRNA against *Sorbs1* submitted to a scratch-wound assay were taken before (oh) and 16 h after making the scratch to quantify their migratory abilities. Scale bar represents 100 μm. Histogram represents mean ± sd of 3 independent experiments (*: *P* < *0.05*, Student’s t test). (D) Rac1 activity in HUVECs transfected with control (Ctl) or *Sorbs1* siRNA was assessed by Western blot analysis of PAK2 and PAK4 phosphorylation using phospho-specific antibodies. Total amounts of PAK2 and PAK4 were used as loading controls. (E) Co-localization of Sorbs1 and adhesion complexes was analyzed in HUVECs by confocal microscopy after immuno-staining using antibodies specific to paxillin and sorbs1. Scale bar represents 20 μm. (F) Adhesion complexes were analyzed by confocal microscopy after immunostaining of paxillin and phospho-paxillin in HUVECs transfected with control or *Sorbs1* siRNA as in Figure 6C. Nascent adhesions (NA) and focal complexes (Fx) were classified into two size categories and their distribution in each condition was quantified (*n* = 21; *: *P* < *0.05*, ns = non-significant, Student’s t test). (G-I) Western blotting was used to assess phosphorylation of Paxillin in Ctl and *Sorbs1*-depleted cells using a phospho-specific antibody (G), Sorbs1 expression after seeding onto fibronectin for the indicated time (H) and FAK-Src-ERK signaling in Ctl and *Sorbs1*-depleted cells using phospho-specific antibodies for activated FAK and ERK and inactivated Src (I). Total amounts of the corresponding proteins or actin level were used as loading controls. (J) WT and *sorbs1*^*−/−*^ embryos were treated or not with RhoA inhibitor at 26 hpf and the number of sprouting cells at the edge of developing CVP at 28 hpf was quantified (*n* = number of embryos; ***P < *0.001*, ns = non-significant; Mann–Whitney U-test).

**Additional file 2:Movie 1.** Time-lapse imaging of secondary venous sprouting in control *Tg(fli1a:eGFP)y1* embryos. Z-series images in the region centered on the yolk extension end using a 3-μm-step confocal based scan were taken every 20 min and showing sprouting of ECs from the PCV and their quick connection to the neighboring aISVs before either fusing with the ISV or stabilizing PLs.

**Additional file 3:Movie 2.** Time-lapse imaging of secondary venous sprouting in sorbs1^*−*/*−*^* Tg(fli1a:eGFP)y1* embryos. Z-series images in the region centered on the yolk extension end using a 3-μm-step confocal based scan were taken every 20 min and showing venous sprouts spending time before connecting to the aISVs.

**Additional file 4: Movie 3.** Time-lapse imaging of secondary venous sprouting in sorbs1^*−*/*−*^* Tg(fli1a:eGFP)y1* embryos. Z-series images in the region centered on the yolk extension end using a 3-μm-step confocal based scan were taken every 20 min and showing very low sprouting activity from the venous cells.

**Additional file 5:** Compilation of all original uncropped blots.

**Additional file 6:** An excel file with all numerical data used in the paper.

## Data Availability

All data and reagents generated in this study will be made available upon request to the lead contact.

## Abbreviations

bp: Base pair
CV: Cardinal vein
CVP: Caudal vein plexus
DA: Dorsal aorta
DLAV: Dorsal longitudinal anastomotic vessel
DLLV: Dorsal longitudinal lymphatic vessels
dpf: Days post-fertilization
EC: Endothelial cells
ECM: Extracellular matrix
ELV: Ectopic longitudinal vessel
EV: Ectopic vessel
FA: Focal adhesions
Fx: Focal complexes
GAP: GTPase-activating protein
GFP: Green fluorescent protein
gRNA: Guide RNA
hpf: Hours post-fertilization
ICV: Interconnecting vessels
ISV: Intersegmental vessel
Mo: Morpholino
NA: Nascent adhesions
PCV: Posterior cardinal vein
PL: Parachordal lymphangioblasts
SIV: Subintestinal vein
SIVP: Subintestinal venous plexus
SoHo: Sorbs homology domain
TD: Thoracic duct
