# Supplementary material for: The cytoskeleton adaptor protein Sorbs1 controls the development of lymphatic and venous vessels in zebrafish
Source: BMC Biol. 2024 Feb 27;22:51. doi: 10.1186/s12915-024-01850-z (PMC10900589; doi:10.1186/s12915-024-01850-z)
Supplement: Supplementary file 1 — Additional file 1:Supplemental Figure 1. Characterization of Sorbs1 depletion in vivo and its expression in endothelial cells. (A) Phylogenetic tree was constructed from human, mouse and potential zebrafish mRNA of Sorbs1, Sorbs2 and Sorbs3 using ClustalW software. One zebrafish ortholog was identified for sorbs1 (Dr sorbs1) and sorbs3 (Dr sorbs3) and two for sorbs2 (Dr sorbs2a and Dr sorbs2b). (B) DNA and amino-acid sequences of the region around the CRISPR/Cas9 target site of the wild-type sorbs1 allele (WT) and its 14-bp deletion (-14) following CRISPR/ Cas9-based editing. (C) Western blotting analysis of protein extracts from wild-type (WT) and Sorbs1 mutant (sorbs1−/−) embryos, using anti-Sorbs1 antibody. GAPDH was used as loading control. (D) Phase-contrast imaging of 48 hpf embryos (upper panel) and zooms on the somites from trunk regions (down panel) of wild-type (WT) and sorbs1−/− Tg(fli1a:eGFP) embryos used to quantify the antero-posterior (AP) body length (white line), the brain size (dashed white line) and the somite angle (white circular arc) and AP length (white line) (n = number of embryos, ns = non-significant, Mann–Whitney U-test). Scale bars represent 500 μm. (E) Expression of Sorbs1 in various human tissues assessed by immunohistochemistry. Typical Sorbs1 staining in endothelial cells is illustrated for the indicated tissues. Boxes correspond to the enlarged area showing expression of Sorbs1 in blood vessels (arrows). Scale bars represent 50 μm and 500 μm respectively in large and zoomed picture. (F) Western blotting analysis of Sorbs1 expression in various human endothelial cells: HDMECs (Human Dermal Microvascular Endothelial Cells), HMECs (Human Mammary Epithelial Cells), HUAECs (Human Umbilical Artery Endothelial Cells), HUVECs (Human Umbilical Endothelial Cells), HMVEC-dLyAd (Human Dermal Lymphatic Microvascular Endothelial Cells), HEK293 (Human Embryonic Kidney 293) and Hela cells. HSP90 was used as a loading control. (G) Whole mount in situ [file 12915_2024_1850_MOESM1_ESM.pdf]

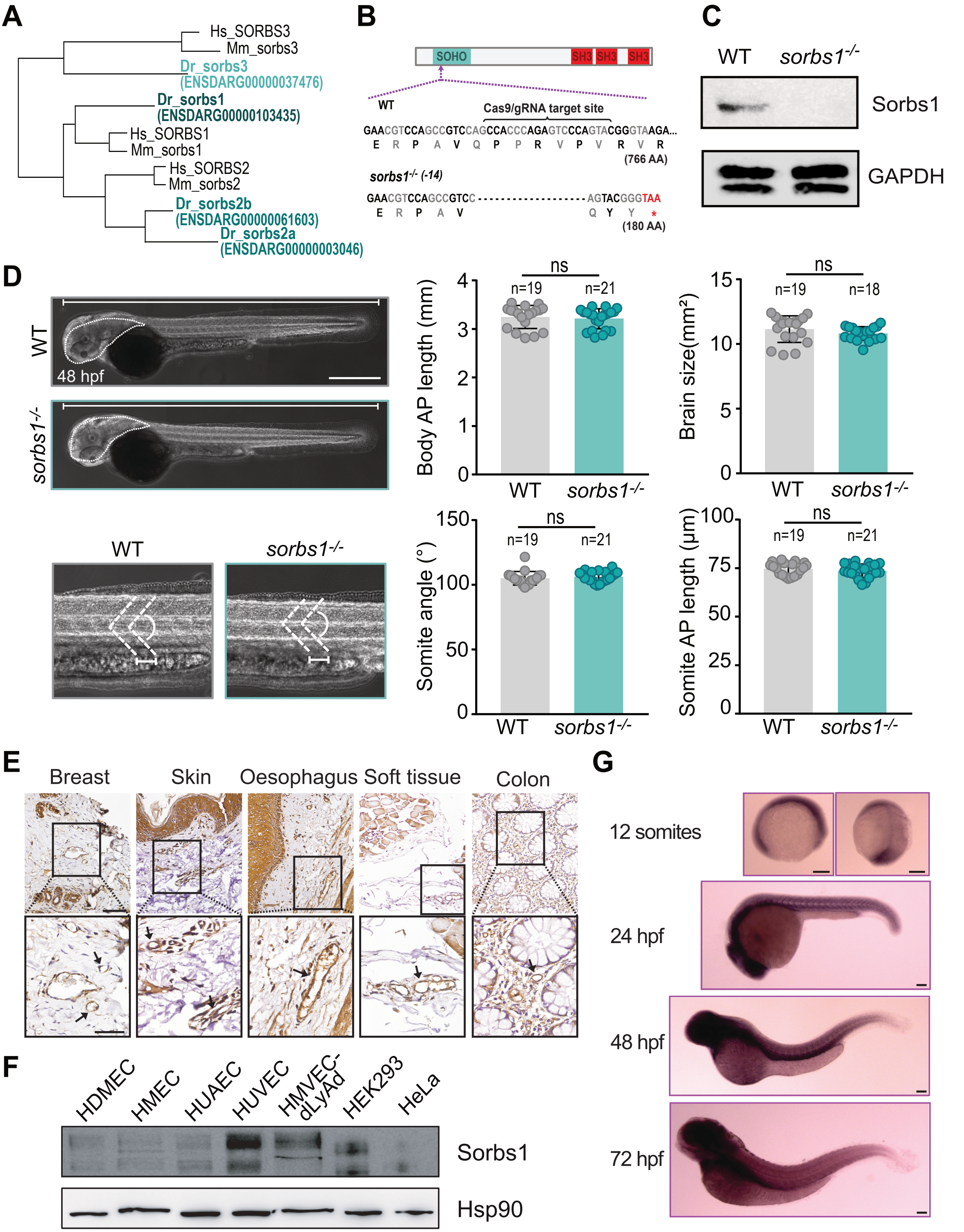


**Supplemental Figure 1**

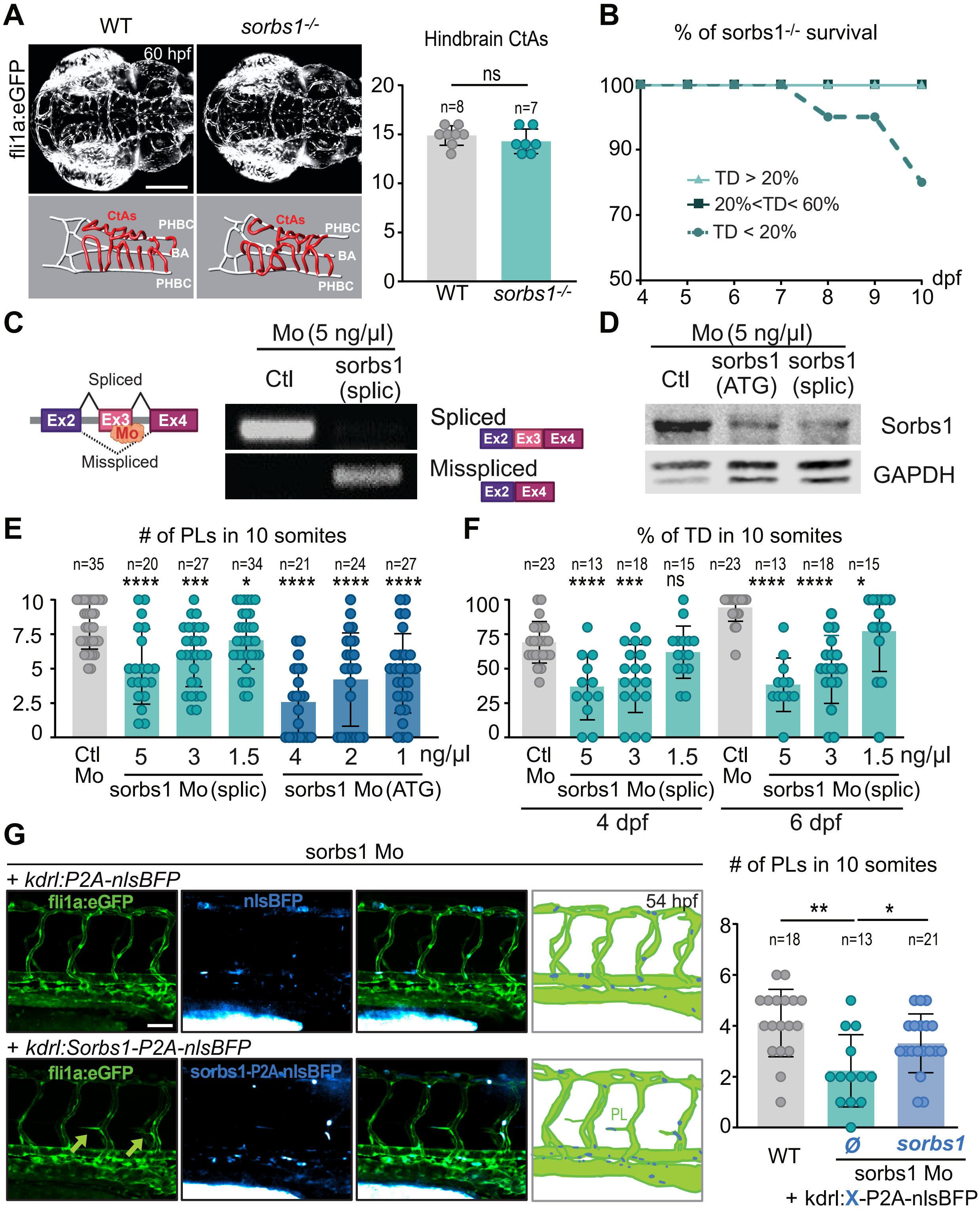


**Supplemental Figure 2**

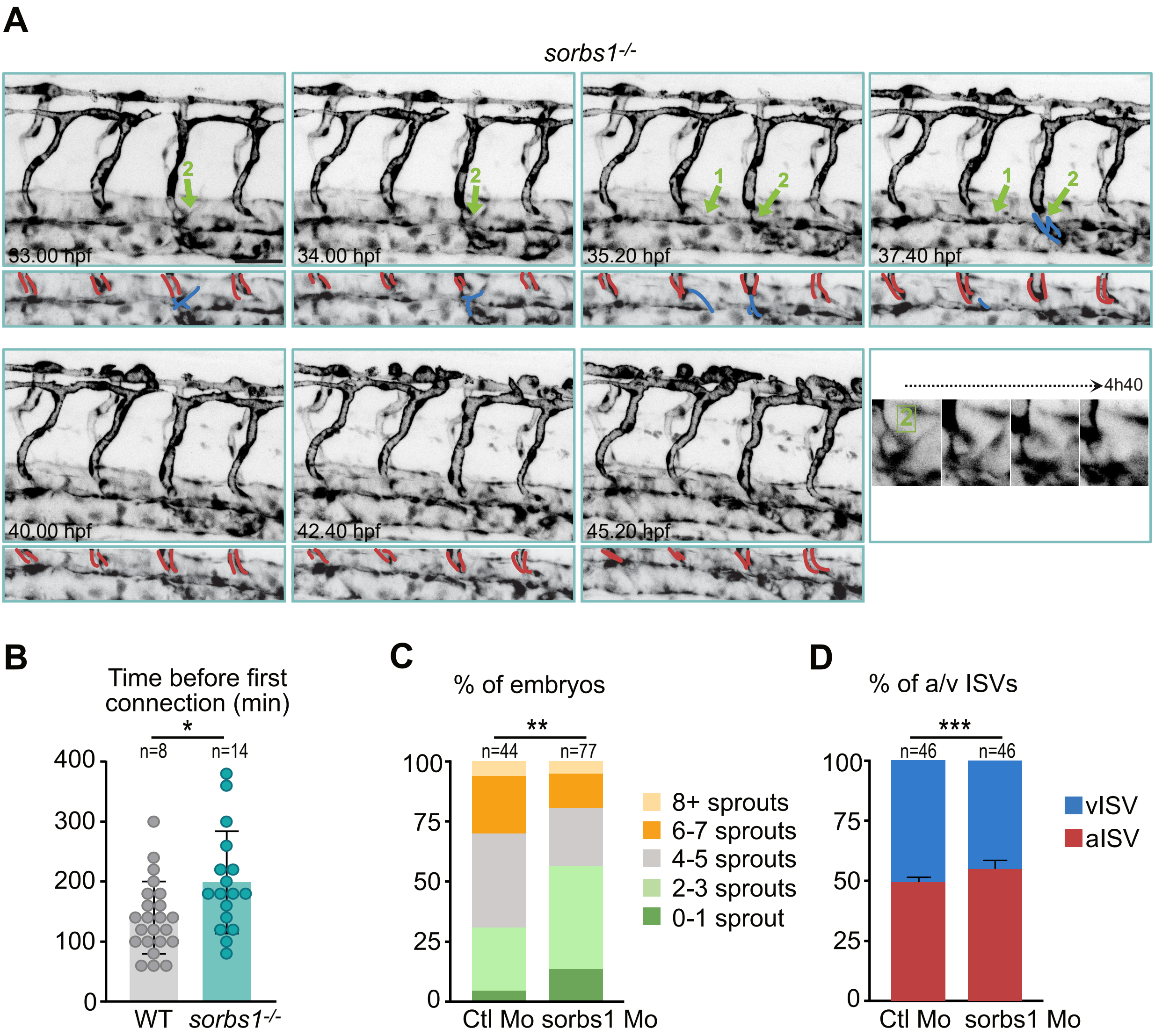


**Supplemental Figure 3**

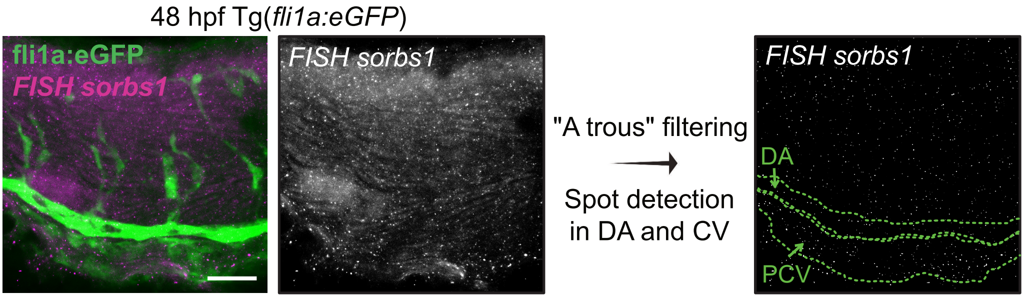


# Supplemental Figure 4

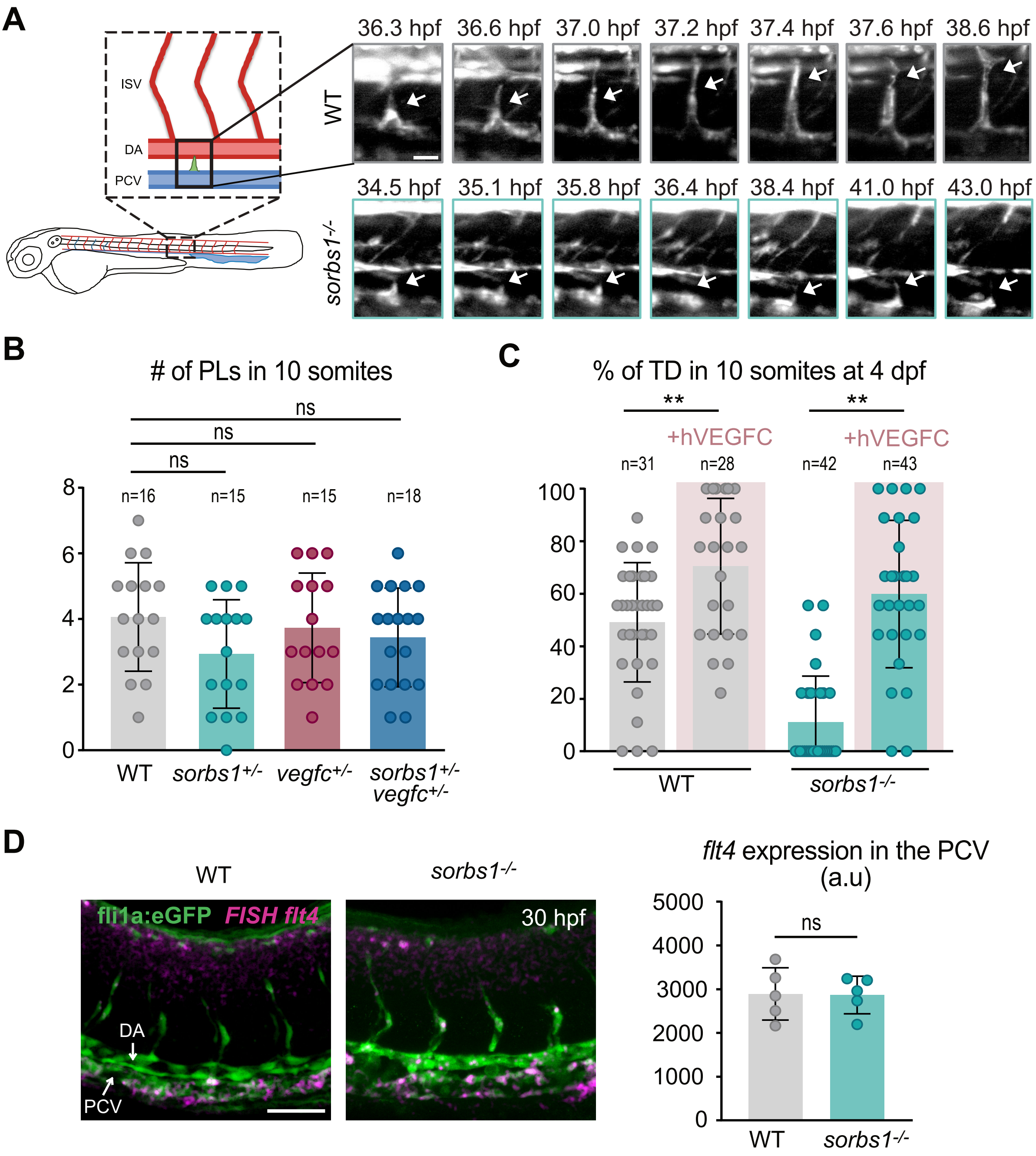


**Supplemental Figure 5**

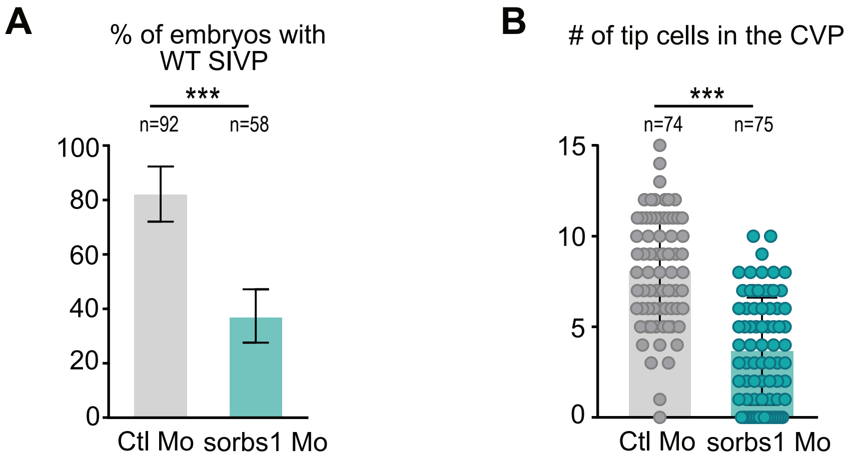


**Supplemental Figure 6**

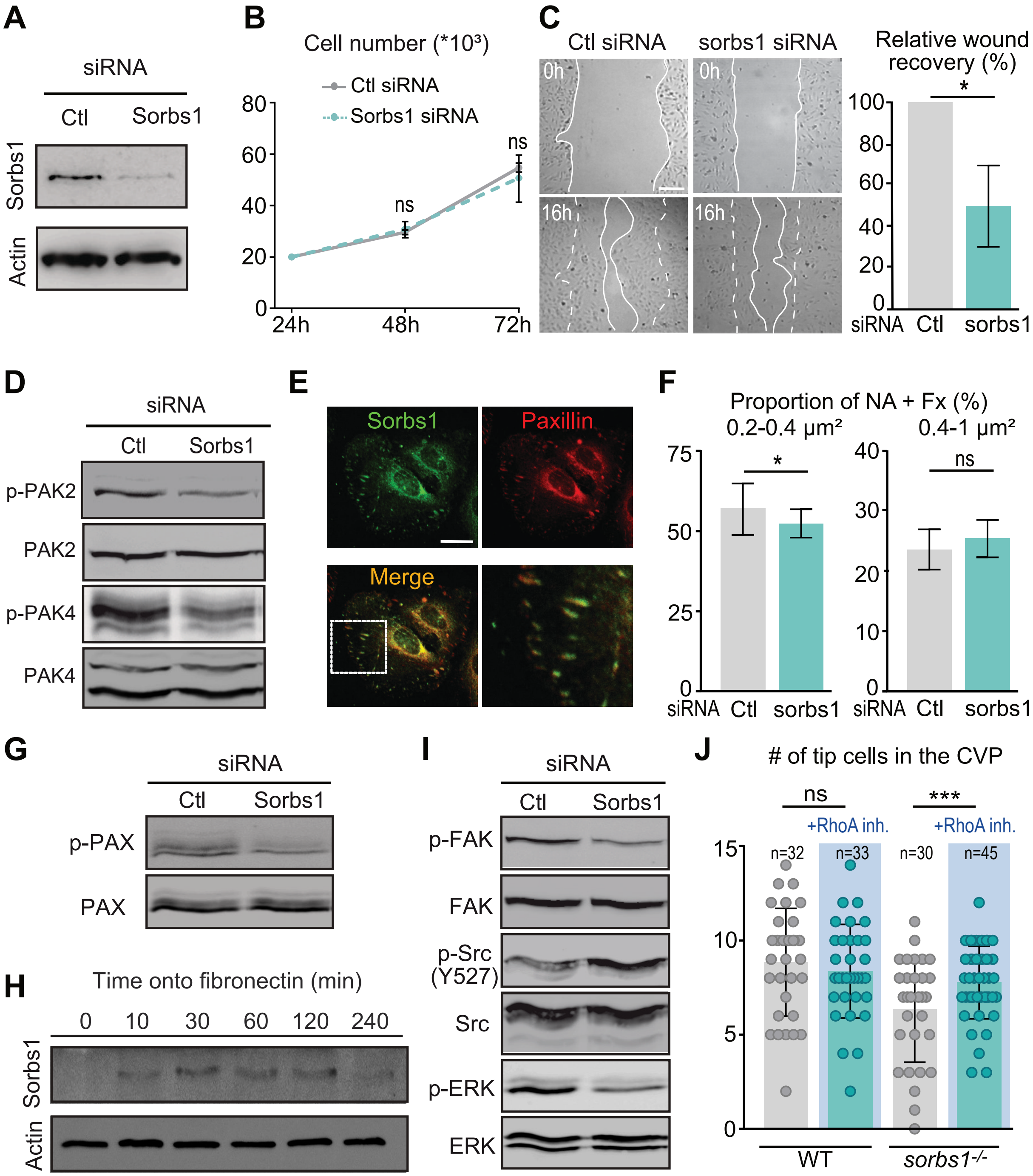


**Supplemental Figure 7**
