# Supplementary figures and images for: The cytoskeleton adaptor protein Sorbs1 controls the development of lymphatic and venous vessels in zebrafish
Source: BMC Biol. 2024 Feb 27;22:51. doi: 10.1186/s12915-024-01850-z (PMC10900589; doi:10.1186/s12915-024-01850-z)

**Figure S1C and S2D**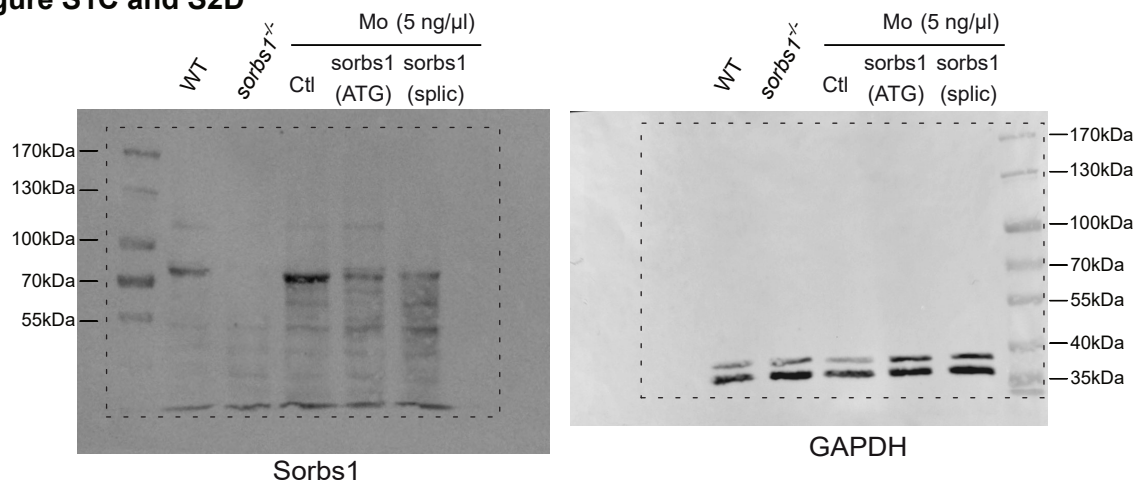**Figure S1F**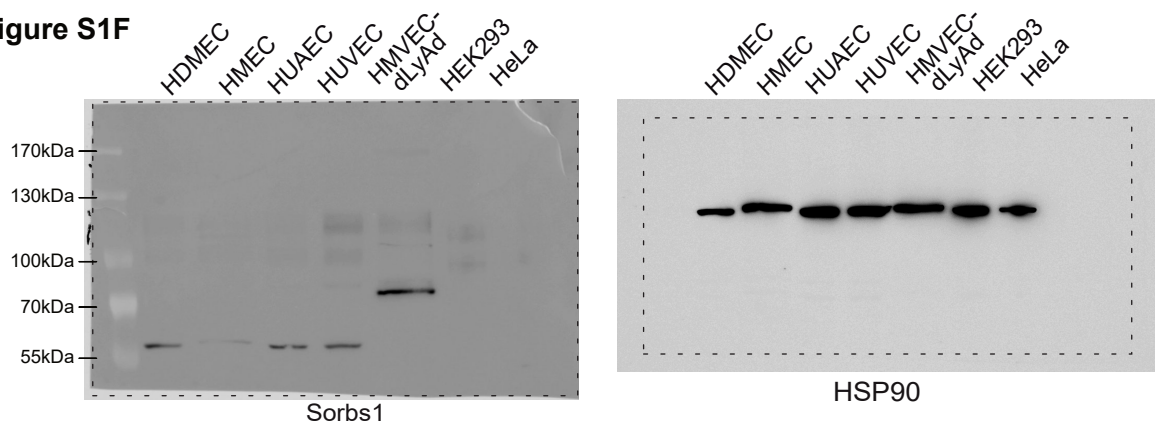**Figure S7A**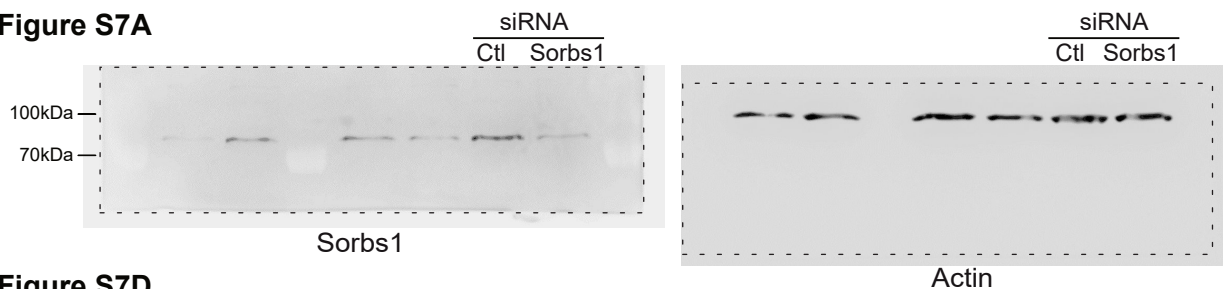**Figure S7D**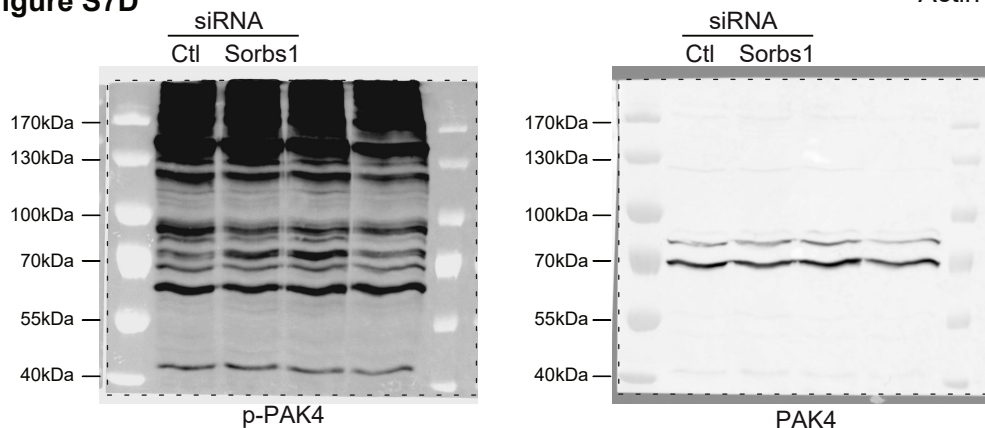

**Figure S7G**

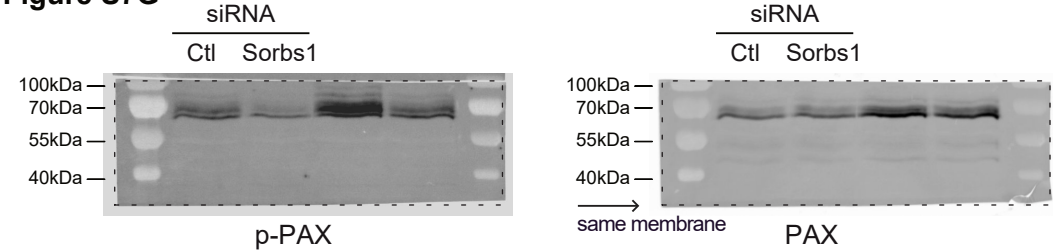

**Figure S7H**

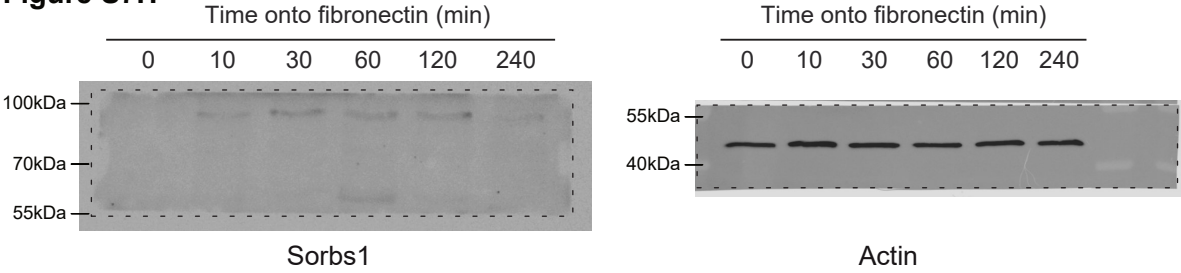

**Figure S7I**

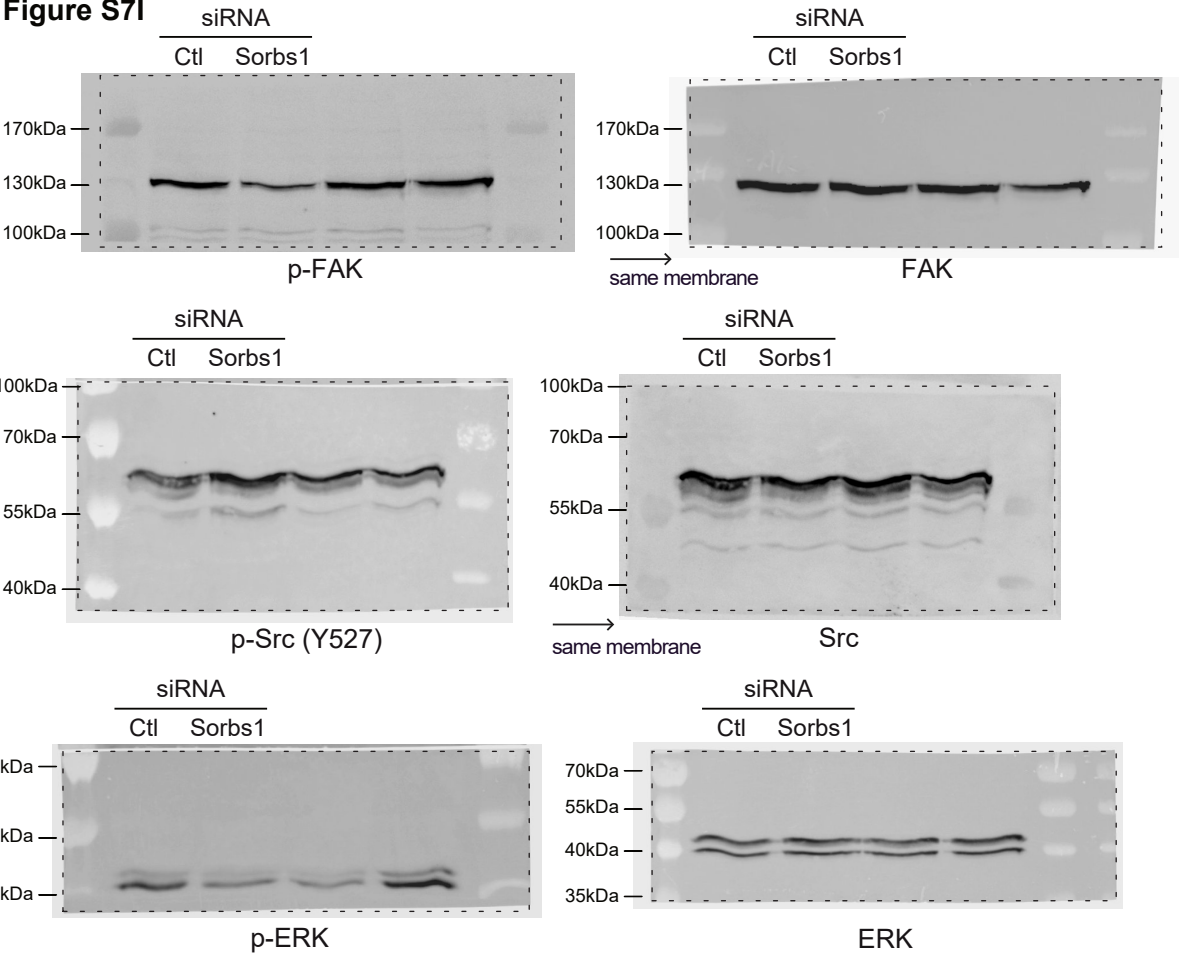

Supplement: Supplementary file 5 — Additional file 5: Compilation of all original uncropped blots. [file 12915_2024_1850_MOESM5_ESM.pdf]
